# Interleukin‐6 initiates muscle‐ and adipose tissue wasting in a novel C57BL/6 model of cancer‐associated cachexia

**DOI:** 10.1002/jcsm.13109

**Published:** 2022-11-09

**Authors:** Isabella Pototschnig, Ursula Feiler, Clemens Diwoky, Paul W. Vesely, Thomas Rauchenwald, Margret Paar, Latifa Bakiri, Laura Pajed, Peter Hofer, Karl Kashofer, Nyamdelger Sukhbaatar, Gabriele Schoiswohl, Thomas Weichhart, Gerald Hoefler, Christoph Bock, Martin Pichler, Erwin F. Wagner, Rudolf Zechner, Martina Schweiger

**Affiliations:** ^1^ Institute of Molecular Biosciences University of Graz Graz Austria; ^2^ Diagnostic and Research Institute of Pathology Medical University of Graz Graz Austria; ^3^ Division of Physiological Chemistry, Otto‐Loewi Research Center Medical University of Graz Graz Austria; ^4^ Department of Laboratory Medicine Medical University of Vienna Vienna Austria; ^5^ Institute of Medical Genetics Medical University of Vienna Vienna Austria; ^6^ Department of Pharmacology and Toxicology University of Graz Graz Austria; ^7^ CeMM Research Center for Molecular Medicine of the Austrian Academy of Sciences Vienna Austria; ^8^ Institute of Artificial Intelligence, Center for Medical Statistics, Informatics, and Intelligent Systems Medical University of Vienna Vienna Austria; ^9^ Division of Oncology Medical University of Graz Austria; ^10^ Department of Dermatology Medical University of Vienna Vienna Austria; ^11^ BioTechMed‐Graz Graz Austria; ^12^ Field of Excellence BioHealth ‐ University of Graz Graz Austria

**Keywords:** Adipose tissue, C57BL/6, Cachexia, Cancer, Interleukin‐6

## Abstract

**Background:**

Cancer‐associated cachexia (CAC) is a wasting syndrome drastically reducing efficacy of chemotherapy and life expectancy of patients. CAC affects up to 80% of cancer patients, yet the mechanisms underlying the disease are not well understood and no approved disease‐specific medication exists. As a multiorgan disorder, CAC can only be studied on an organismal level. To cover the diverse aetiologies of CAC, researchers rely on the availability of a multifaceted pool of cancer models with varying degrees of cachexia symptoms. So far, no tumour model syngeneic to C57BL/6 mice exists that allows direct comparison between cachexigenic‐ and non‐cachexigenic tumours.

**Methods:**

MCA207 and CHX207 fibrosarcoma cells were intramuscularly implanted into male or female, 10–11‐week‐old C57BL/6J mice. Tumour tissues were subjected to magnetic resonance imaging, immunohistochemical‐, and transcriptomic analysis. Mice were analysed for tumour growth, body weight and ‐composition, food‐ and water intake, locomotor activity, O_2_ consumption, CO_2_ production, circulating blood cells, metabolites, and tumourkines. Mice were sacrificed with same tumour weights in all groups. Adipose tissues were examined using high‐resolution respirometry, lipolysis measurements *in vitro* and *ex vivo*, and radioactive tracer studies *in vivo*. Gene expression was determined in adipose‐ and muscle tissues by quantitative PCR and Western blotting analyses. Muscles and cultured myotubes were analysed histologically and by immunofluorescence microscopy for myofibre cross sectional area and myofibre diameter, respectively. Interleukin‐6 (*Il‐6*) was deleted from cancer cells using CRISPR/Cas9 mediated gene editing.

**Results:**

CHX207, but not MCA207‐tumour‐bearing mice exhibited major clinical features of CAC, including systemic inflammation, increased plasma IL‐6 concentrations (190 pg/mL, *P* ≤ 0.0001), increased energy expenditure (+28%, *P* ≤ 0.01), adipose tissue loss (−47%, *P* ≤ 0.0001), skeletal muscle wasting (−18%, *P* ≤ 0.001), and body weight reduction (−13%, *P* ≤ 0.01) 13 days after cancer cell inoculation. Adipose tissue loss resulted from reduced lipid uptake and ‐synthesis combined with increased lipolysis but was not associated with elevated beta‐adrenergic signalling or adipose tissue browning. Muscle atrophy was evident by reduced myofibre cross sectional area (−21.8%, *P* ≤ 0.001), increased catabolic‐ and reduced anabolic signalling. Deletion of IL‐6 from CHX207 cancer cells completely protected CHX207^IL6KO^‐tumour‐bearing mice from CAC.

**Conclusions:**

In this study, we present CHX207 fibrosarcoma cells as a novel tool to investigate the mediators and metabolic consequences of CAC in C57BL/6 mice in comparison to non‐cachectic MCA207‐tumour‐bearing mice. IL‐6 represents an essential trigger for CAC development in CHX207‐tumour‐bearing mice.

## Introduction

Cancer‐associated cachexia (CAC) is a progressive, multifactorial metabolic wasting syndrome that affects up to 80% of cancer patients and drastically reduces treatment options and survival rates of affected individuals.^1^ In CAC, a chronic metabolic imbalance favouring catabolism over anabolism^2^ results in adipose tissue‐ and skeletal muscle wasting that cannot be compensated by nutritional measures.^1^

Catabolic reprogramming in muscle includes increased protein degradation, reduced protein synthesis, and impaired regeneration, leading to a progressive loss of skeletal muscle mass.^3^ In white adipose tissue (WAT), elevated lipolysis, reduced adipo‐lipogenesis,^4^ and upregulation of energy dissipating processes, such as non‐shivering thermogenesis contribute to fat loss.^5^, ^6^

Systemic inflammation and catabolic transformation are triggered by numerous circulating proinflammatory and/or catabolic signalling molecules with pleiotropic effects in different tissues,^7^ complicating mechanistic studies and the development of drugs to treat CAC. The complexity of CAC as a ‘systemic disease’ also implies that it can only be studied on an organismal level. Since human studies are difficult due to ethical issues, the investigation of molecular mechanisms underlying CAC mostly depends on animal studies in appropriate models of the human disease.

CAC research in mice predominantly focuses on three basic tumour models: patient‐derived tumour xenografts, genetically engineered mouse models (GEMMs), and syngeneic allografts, which have been summarized and compared before.^8^ All have been proven valuable for cachexia research, albeit with limitations, such as metabolic alterations due to genetic background,^9^ variability of disease onset and progression, tissue infiltration of the primary tumour, or metastases.^8^, ^10^ Syngeneic models, generated by injecting murine cancer cells into immunocompetent mice, undergo synchronized and reproducible tumour growth as well as cachexia onset and development within a consistent time‐period. For studying CAC in C57BL/6 mice, Lewis lung carcinoma (LLC) and B16F10 melanoma cells are the most used syngeneic allografts. However, their inherent genomic instability caused the emergence of many heterogenous subclones ranging from low to high tumour burden and different degrees of cachexia severity^5^ ^[^ ^S1,S2^ ^]^ Moreover, no suitable non‐cachexigenic cancer cell lines exist for these models.^8^ Given the complexity and variability of CAC, there is an urgent demand for additional well‐characterized animal models to study the disease.

Here, we introduce a murine fibrosarcoma model for studying CAC in C57BL/6 mice. Cachexigenic CHX207 cells evolved from non‐cachexigenic 3‐methyl‐cholanthrene (MCA)‐induced fibrosarcoma 207 cells allowing the comparison of cachectic‐ and non‐cachectic mice bearing the same tumour type. Cachexia in CHX207 mice manifests with skeletal muscle‐ and adipose tissue wasting accompanied by altered systemic energy metabolism and inflammation.

## Methods

### Cell culture

MCA207 and CHX207 fibrosarcoma cells were cultured and analysed for proliferation and by Next‐generation sequencing as described in the Supplemental Methods. CRISPR/Cas9‐mediated *Il6* gene deletion in MCA207 and CHX207 fibrosarcoma cells was performed as described in the Supplemental Methods. C2C12 myoblasts were differentiated into myotubes, treated with cancer‐cell conditioned medium, immunolabeled, and diameters analysed as described in the Supplemental Methods.

### Animal studies

Ten‐ to 11‐week‐old C57Bl/6J mice were injected with 1 × 10^6^ cancer cells or 1× PBS as vehicle control into the musculus gastrocnemius of the right hind leg. At study endpoints blood was drawn via the retro‐orbital plexus, mice were sacrificed by cervical disocation, and tissues excised and snap‐frozen in liquid nitrogen. *In vivo* body‐ and tumour composition was assessed using NMR and MRI, respectively (Supplemental Methods). Body temperature was assessed using a rectal probe (Physitemp, NJ, USA). Systemic metabolism was analysed using a laboratory animal monitoring system as described (Supplemental Methods).

### Transcriptomic analysis

For transcriptomic analysis using the Illumina HiSeq 3000/4000 platform, RNA was isolated from tumour tissue and analysed as described in the Supplemental Methods.

### Lipid metabolism

*In vivo* radioactive tracer studies were performed using ^14^C bromo‐palmitic acid and tissue homogenates were subjected to high‐resolution respirometry as described in the Supplemental Methods. The release of glycerol from gWAT explants, *in vitro* TG hydrolase activities and tissue acylglycerol content were measured as described in the Supplemental Methods.

### Immune‐histochemical and Western blotting analyses

Tissues were excised, washed in 1× PBS, fixed in 4% buffered formaldehyde, and embedded in paraffin. Paraffin‐sections were incubated with anti‐CD31 antibody or with haematoxylin and eosin. Myofibre cross sectional area of muscle was determined as described in the Supplemental Methods. For protein expression analysis, tissues were disrupted in ice‐cold solution A; 10 μg protein were subjected to SDS‐PAGE and Western blotting analysis as described in the Supplemental Methods.

### RNA isolation and real‐time (RT) qPCR

Total tissue RNA was extracted using TRIzol (Invitrogen, Waltham, USA) and cDNA was prepared using Luna Script RT Supermix Kit (NEB) according to the manufacturer's instructions. RT‐qPCR was performed using StepOnePlus™ RT‐PCR System (Thermo Fisher Scientific) with SYBR green (Bio‐Rad Laboratories) and appropriate primers (Supplemental Methods).

### Statistical analysis

Data are shown as means with standard deviations (SD). Statistical significances were determined by two‐sided Student's *t*‐test, one‐way ANOVA followed by Tukey's *post hoc* analysis or ANCOVA analysis (*P ≤ 0.05, **P ≤ 0.01, ***P ≤ 0.001, ****P ≤ 0.0001) using GraphPad Prism 8.0.1. Group size estimations were based upon a power calculation to minimally yield an 80% chance to detect a significant difference of *P* < 0.05 between groups.

## Results

### A subclone derived from MCA207 fibrosarcoma induces cachexia in male and female C57BL/6J mice

**Figure 1:**
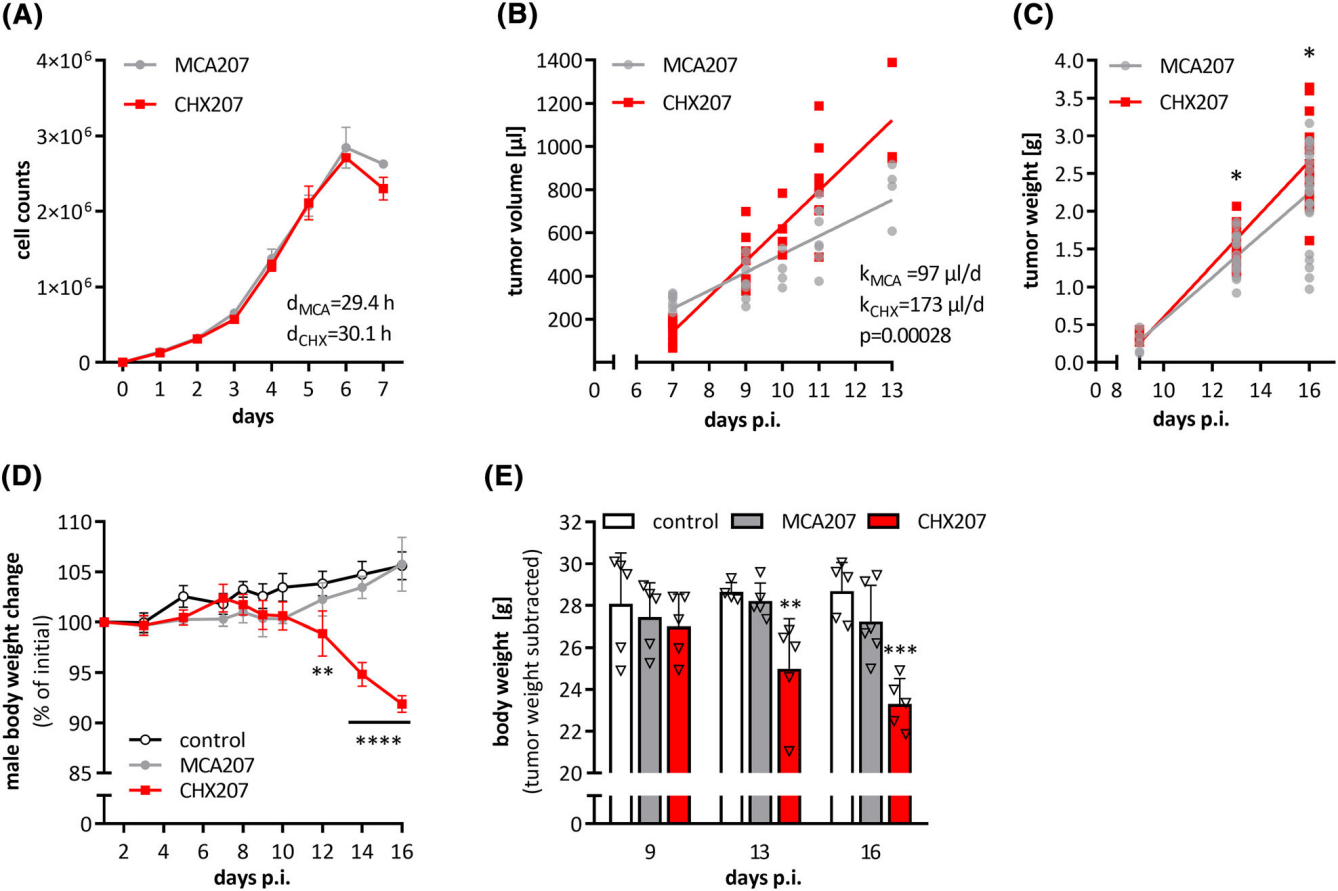
CHX207 fibrosarcoma causes progressive body weight loss in C57BL/6J mice. (A) Proliferation of MCA207 and CHX207 cells in culture was determined by counting cells on 7 consecutive days after seeding (*n* = 3, d = doubling time). (B–E) Ten‐ to 11‐week‐old male C57BL/6J mice were injected with 1 × 10^6^ MCA207, 1 × 10^6^ CHX207 cells, or 1xPBS (control). (B) Tumour volume of MCA207‐ and CHX207‐tumour‐bearing mice was assessed by MR imaging at the indicated time points. (C) Mice were sacrificed, tumours were excised and weighed at Day 9, Day 13 or Day 16 p.i. (*n* = 10–14). (D) Body weight relative to initial body weight of male control‐ and tumour‐bearing mice (*n* = 4–6). (E) Tumour‐free body weight of male control‐ and tumour‐bearing mice (*n* = 4–6). Data are presented as means ± SD. Significance was determined by A,B,C) two‐sided Student’s *t*‐test or D,E) one‐way ANOVA followed by Tukey's *post hoc* analysis (*P ≤ 0.05, **P ≤ 0.01, ***P ≤ 0.001, ****P ≤ 0.0001).

MCA‐induced fibrosarcoma 207 is a well‐established soft tissue sarcoma model that is used to study tumour regression and immunity in C57BL/6 mice.^11^, ^12^ ^[^ ^S3^ ^]^ Continuous passaging of MCA207 cells caused a ‘drift’ in tumour phenotype and cachexia in CHX207‐tumour‐bearing mice. Intramuscular injection of MCA207‐ or CHX207 cells resulted in solid tumours, which were palpable around 7 days post injection (p.i.). Although cancer cell proliferation was not different between MCA207 and CHX207 cells *in vitro* (Figure 1A), we found significantly faster growth of CHX207‐ compared with MCA207 tumours *in vivo* (Figure 1B). After 16 days p.i. MCA207‐ and CHX207 tumours weighed 2.2 ± 0.6 g and 2.7 ± 0.7 g (*P* = 0.02), respectively (Figure 1C).

Intratumoural bleeding and concomitant stretching of the surrounding tissue occurs in 64% of patients with soft tissue sarcoma.^13^ In murine CHX207 tumours, we found an increased transversal tissue relaxation rate R2* by MRI, indicating the emergence of acute tumour hematomas between Day 7 and Day 13 p.i. (Figure S1A). MRI analysis depicted large hemorrhagic lesions in CHX207 tumours at Day 9 p.i. which were negligible in MCA207 tumours (Figure S1B). Haemorrhage often occurs in highly vascularized tumours with dilated, thin‐walled vessels.^14^ Indeed, the endothelial cell marker CD31 was more abundant in CHX207‐ compared with MCA207 tumour sections, indicating higher vascularization of CHX207 tumours (Figure S1C).

The most common distant metastasis in patients with primary fibrosarcoma is observed in the lung.^15^ Similarly, histological analysis of murine whole lung cross sections revealed micro metastases (approx. 0.2 mm) in one out of three CHX207‐ and one out of four MCA207‐tumour‐bearing mice (hereinafter referred to as CHX207 mice and MCA207 mice) (Figure S1D) at Day 13 p.i. indicating no apparent differences in metastasis formation between the two tumour types.

According to the COSMIC database, the top mutated gene in fibrosarcoma is tumour protein p53 (*TP53*). Using next‐generation sequencing, we detected a mutation resulting in an amino acid substitution TP53‐V170L with a pathogenic score of ≥0.99 (functional analysis through hidden Markov models) in MCA207 and CHX207 cells. Phosphatidylinositol 3‐kinase (*Pi3kca*) oncogene mutations (V243F and G1009E) were detected with a pathogenic score ≥0.97 in both cell clones. Using RNA‐sequencing analyses we identified 911 genes downregulated and 1084 genes upregulated at Day 9 p.i. and 1039 genes downregulated and 1136 genes upregulated at Day 12 p.i. in CHX207 tumours compared with MCA207 tumours (Table S2). Differentially expressed genes (DEGs) between MCA207‐ and CHX207 tumours are depicted in volcano plots shown in Figure S1E.

A significant reduction in body weight was observed from Day 12 p.i. in male (Figure 1D) and Day 14 p.i. in female (Figure S1F) CHX207‐ but not MCA207 mice despite an increase in tumour weight (Figure 1C). After 16 days p.i. male CHX207 mice had 3.4 g less tumour‐free body weight than control mice (Figure 1E). MCA207 mice had slightly but not significantly lower tumour‐free body weight compared with control mice (Figures 1E). After 15 days p.i., and with same tumour size as males (Figure S1G), tumour‐free body weight of female CHX207 mice was 4 g lower‐, while body weight of female MCA207 mice was not significantly lower compared with control mice (Figure S1H). These initial data showed that CHX207 but not MCA207 tumours cause progressive cachexia in male and female C57BL/6J mice. As body weight changes were linearly correlated to tumour size (Figure S1I) and to exclude tumour size as a confounder for mechanistic studies on cachexia, we sacrificed mice not always at the same day after cell injection but with same tumour size.

### CHX207 mice are anorectic, have increased resting energy expenditure, and altered circulating metabolites

**Figure 2:**
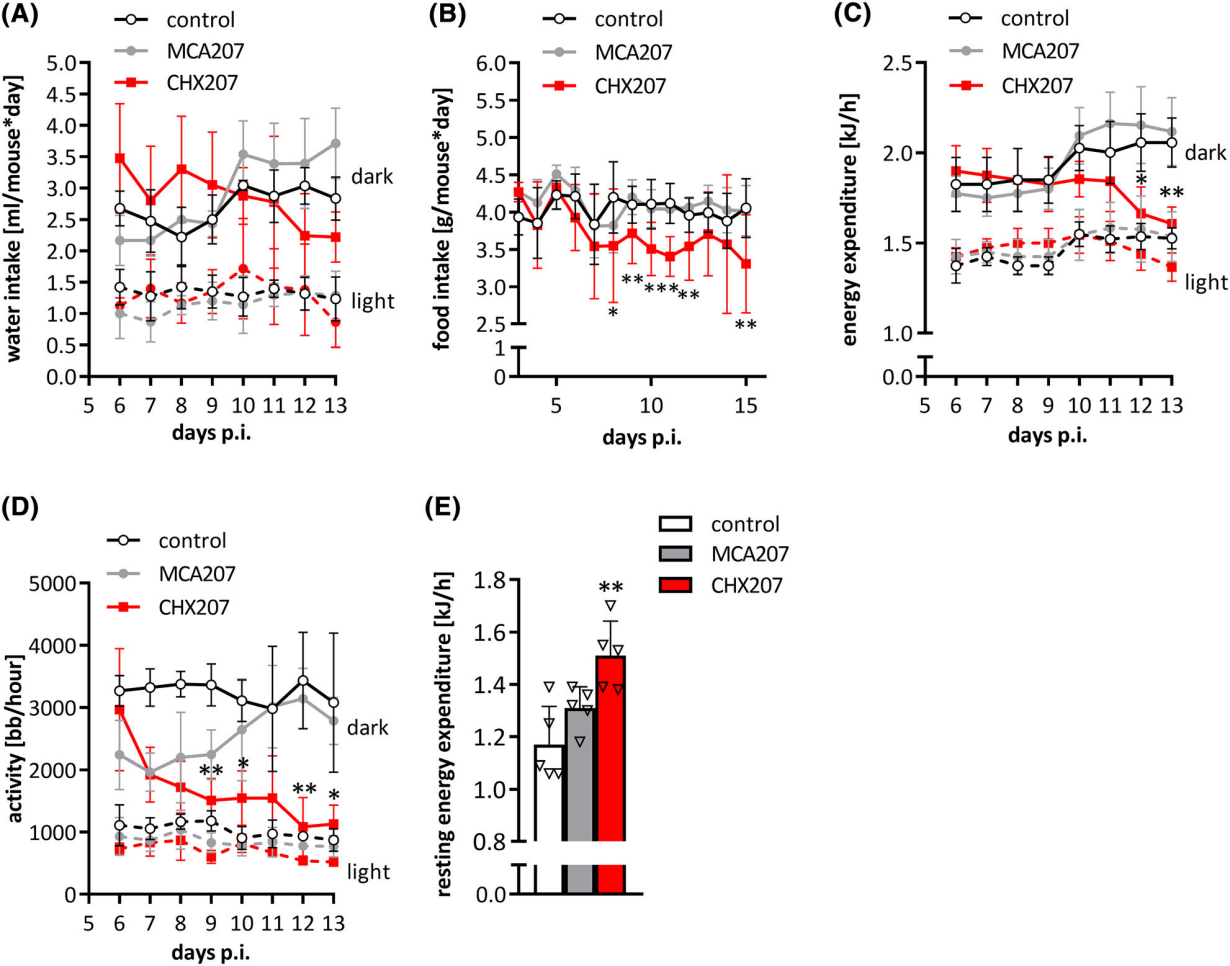
Cachectic CHX207 mice show reduced activity, but higher resting energy expenditure. (A–E) Ten‐ to 11‐week‐old male C57BL/6J mice were injected with 1 × 10^6^ MCA207 cells, 1 × 10^6^ CHX207 cells, or 1× PBS (control) and were analysed using a laboratory animal monitoring system (PhenoMaster, TSE systems GmbH) for 4 consecutive days (dark and light cycles separated) either Days 6–9 or Days 10–13 p.i. for (A) water intake, (C) energy expenditure, or (D) locomotor activity. (B) Food intake was measured by manually weighing food pellets of single housed mice on 15 consecutive days. (E) Resting energy expenditure was determined of anaesthetized mice on Day 13 p.i. in a laboratory animal monitoring system (measurement for 90 min). Data are presented as means ± SD. Significance was determined by one‐way ANOVA followed by Tukey's *post hoc* analysis (*n* = 4–5, *P ≤ 0.05, **P ≤ 0.01).

Anorexia, reduced physical activity, and increased energy expenditure (EE) are commonly observed in mouse models and patients suffering from CAC.^1^, ^2^ To analyse these parameters, we kept control‐, MCA207‐, and CHX207 mice in metabolic cages either from Day 6 to Day 9 p.i. (pre‐cachexia) or from Day 10 to Day 13 p.i. (cachexia). CHX207 mice tended to drink less and exhibited averagely 15% reduced food intake in the cachectic phase compared with control‐ and MCA207 mice (Figures 2A,B). EE was significantly lower (−21%) during dark phases of Day 12 and 13 p.i. in CHX207 mice compared with control and MCA207 mice (Figure 2C). Lower EE significantly correlated with reduced locomotor activity of CHX207 mice (−64% during dark‐ and −33% during light phases) (Figure 2D). Linear regression coefficients for EE and physical activity were 13% and 8% higher in CHX207 mice than in control‐ and MCA207 mice, respectively (Figures S2A,B). To experimentally validate these results, we determined resting EE in sedated mice, which was 29% higher in CHX207 mice than in control mice (Figure 2E). EE of MCA207 mice was not different to control mice.

To investigate whether altered systemic metabolism affects the concentration of circulating metabolites, we performed targeted metabolomics of murine plasma. Fatty acids (FAs), glycerol, triacylglycerol (TG), lactate, glucose, amino acids, and albumin concentrations in plasma did not differ between control‐, MCA207‐, and CHX207 mice (Table S1). In accordance with previous reports on cachectic mice and humans,^16^, ^17^ carnitine and total circulating phosphatidylcholine (PC) levels were reduced by 22% and 21%, respectively, whereas total sphingomyelins (SMs) and SM‐16:0 were 1.2‐fold and 1.6‐fold higher in plasma of CHX207 mice than in MCA207 mice, respectively (Figure S2C,D).

### CHX207‐induced skeletal muscle atrophy results from reduced anabolic and increased catabolic signalling

**Figure 3:**
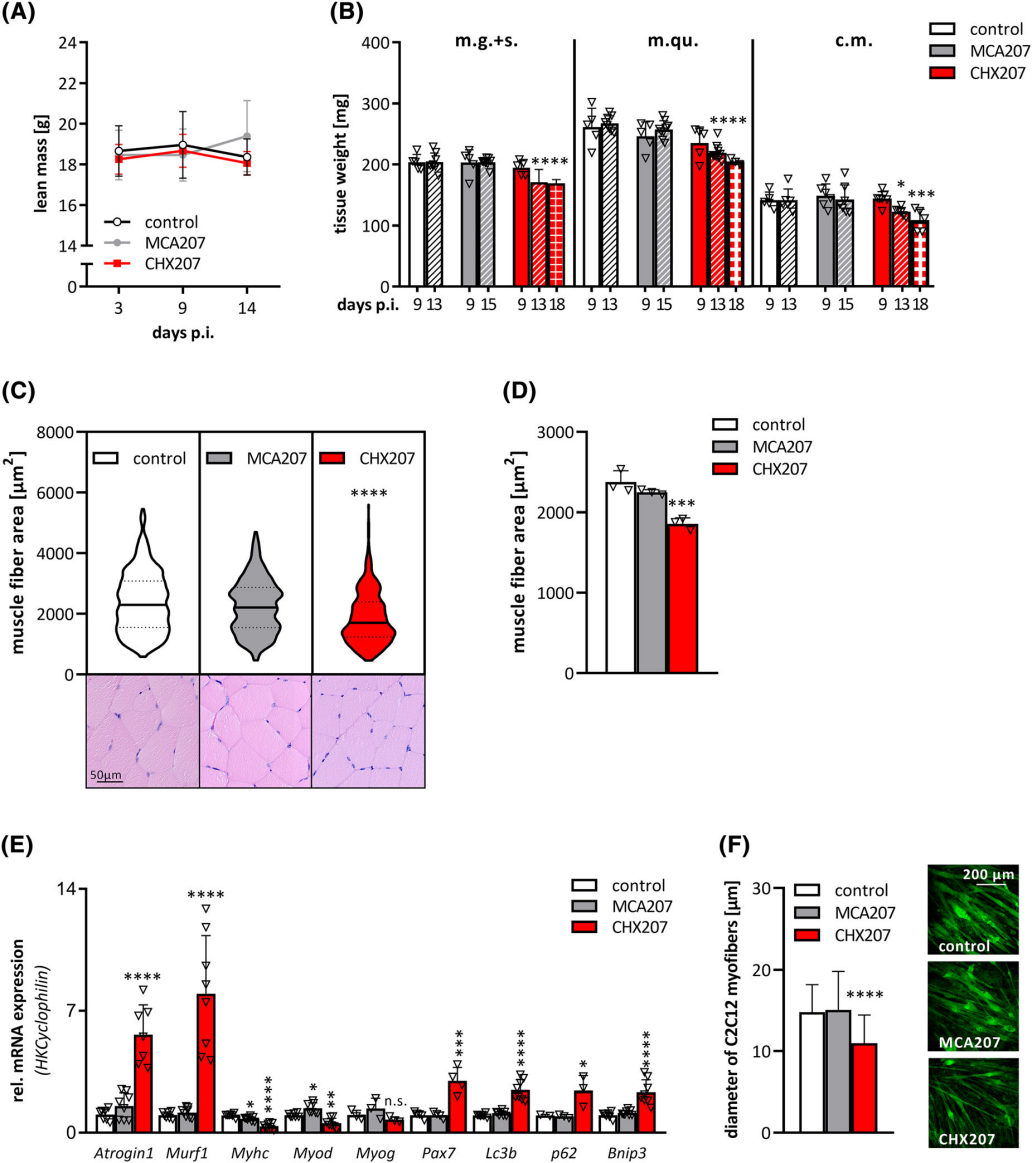
CHX207‐induced skeletal muscle wasting results from reduced anabolic and increased catabolic signalling. (A–E) Ten‐ to 11‐week‐old male C57BL/6J mice were injected with 1 × 10^6^ MCA207 cells, 1 × 10^6^ CHX207 cells, or 1xPBS (control). (A) Total lean mass was determined by NMR (*n* = 10–14). (B) Mice were sacrificed with same tumour size and musculus gastrocnemius + soleus (m.g. + s.), musculus quadriceps (m.qu.) and cardiac muscle (c.m.) were excised and weighed (Day 9 p.i., *n* = 5; Day 13 or 15 p.i., *n* = 8–10; Day 18 p.i., *n* = 3). (C,D) muscle fibre areas (CSA) were measured on H&E‐stained cross‐sections of m.qu. Using CaseViewer (Day 18 p.i.; *n* = 3; >140 fibres per muscle). (C) Violin plot and representative histological images and (D) means of CSA. Each dot represents the mean of >140 CSA of one m.qu. (E) mRNA expression levels of marker genes for muscle catabolic signalling (*Atrogin*, *Murf1)*, muscle protein synthesis/myogenic differentiation (*Myhc*, *Myod*, *Myogenin*, *Pax7*) and autophagy/apoptosis (*Lc3b*, *p62*, *Bnip3)* in m.qu. were determined by qRT‐PCR. *Cyclophilin* was used as housekeeping gene (Day 13 p.i., *n* = 4–8). (F) Differentiated C2C12 myotubes were incubated with control‐, 10% MCA207‐ or CHX207‐cancer cell conditioned medium for 48 h. Myotubes were visualized using Actinin antibody and confocal microscopy. Diameters of approximately 10 myotubes per field and 20 fields per condition were measured using Fiji. Data are presented as means ± SD. Significance was determined by one‐way ANOVA followed by Tukey's *post hoc* analysis (*n* = 4–6, *P ≤ 0.05, **P ≤ 0.01, ***P ≤ 0.001, ****P ≤ 0.0001).

*In vivo* NMR analyses revealed a trend toward increased lean body mass in MCA207 mice 14 days p.i., presumably due to an increase in tumour weight (Figures 3A and 1B). In contrast, lean body mass of CHX207 mice did not increase despite tumour growth (Figure 3A). Accordingly, skeletal muscle and cardiac muscle weights were significantly reduced in male and female CHX207 mice compared with MCA207 mice with same tumour size (Figures 3B and S3A). Musculus gastrocnemius + soleus (m.g. + s.) was reduced by 16% in CHX207 mice of both sexes whereas m.quadriceps (m.qu.) and cardiac muscle (c.m.) were more severely reduced in female CHX207 mice (−27% and −23%, respectively) compared with male CHX207 mice (−18%, and −15%, respectively), relative to sex‐matched control mice (Figure S3A). At a later timepoint (18 days p.i.) m.g. + s., m.qu., and c.m. weighed 17%, 23%, and 23% less in male CHX207 mice than in control mice (Figure 3B).

Histological analyses of m.qu. revealed reduced myofibre cross sectional area in CHX207‐ compared with control mice (−21.8%) and MCA207 mice (−17.4%), respectively (Figure 3C,D). Muscle acylglycerol content was not significantly different between control, MCA‐, and CHX207 mice 13 days p.i., suggesting no apparent myosteatosis (Figure S3B). Plasma creatine kinase (CK) activity, which is a measure for skeletal muscle damage, was increased 2.8‐fold and 1.8‐fold in CHX207‐ compared with MCA207‐ and control mice, respectively (Figure S3C). Thirteen days p.i., mRNA expression of *Atrogin‐1* and muscle RING‐finger protein‐1 (*Murf‐1*), two major E3 ubiquitin ligases ubiquitinylating and thereby marking proteins for proteasomal degradation, were increased 4.4‐fold and 6‐fold, respectively, in skeletal muscles of CHX207 mice compared with control mice (Figure 3E). Concomitantly, K48‐linked polyubiquitylation of proteins was 1.8‐fold and 2‐fold increased in m.qu. of CHX207 mice compared with MCA207‐ and control mice (Figure S3D), respectively, indicating increased proteasomal degradation. Additionally, BCL2/adenovirus E1B 19 kDa protein‐interacting protein 3 (*Bnip3*) mRNA was increased 2.3‐fold (Figure 3E) and protein abundance of caspase 3 was increased 7.1‐fold and 3.8‐fold (Figure S3E) in m.qu. of CHX207 mice compared with MCA207‐ and control mice, indicating elevated apoptosis. Finally, muscles of CHX207 mice exhibited increased mRNA expression of ubiquitin‐binding protein *p62* (2.4‐fold) and microtubule‐associated proteins 1A/1B light chain 3B (*Lc3b*, 2.5‐fold) as well as elevated LC3BI protein (2.9‐fold and 9.4‐fold increased), and LC3BII protein (1.2‐fold and 10.2‐fold increased) compared with muscles of MCA207‐ and control mice, respectively, indicative for increased autophagy (Figures 3E and S3D). Along with 3‐fold increased paired box protein‐7 (*Pax7*), reduced mRNA expression of myoblast determination protein 1 (*Myod*) (−44%) and myosin heavy chain (*Myhc*) (−66%) point toward reduced myogenic differentiation in CHX207‐, compared with control mice (Figure 3E). In contrast, no significant difference in the expression of these genes was observed comparing muscles of MCA207 and control mice.

To investigate whether muscle wasting was directly caused by cancer cell‐secreted factors, we treated differentiated C2C12 myotubes with conditioned medium derived from MCA207‐ or CHX207 cells and determined myofibre diameters. CHX207‐medium‐treated myotubes were 25.7% smaller than MCA207‐ or control‐medium‐treated myotubes, indicating a detrimental effect of CHX207‐cell‐derived factors on myotubes (Figure 3F). Therefore, we conclude that reduction of skeletal muscle mass in CHX207 mice is presumably caused by cancer cell‐secreted factors that induce catabolic‐ and reduce anabolic signals.

### Reduced adipo‐lipogenesis and increased lipolysis, but not browning of WAT contribute to adipose tissue loss in CHX207 mice

**Figure 4:**
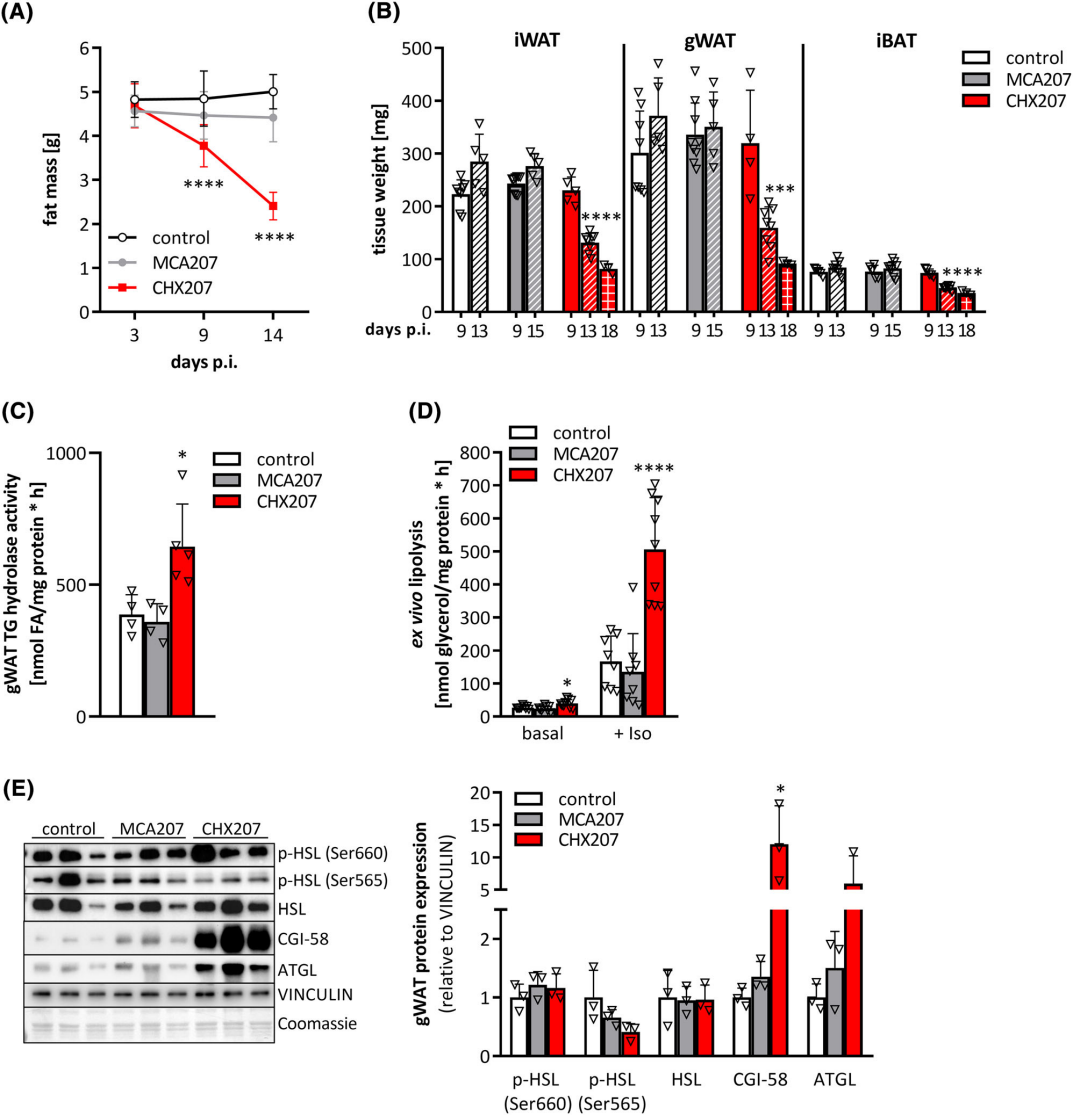
Adipose tissue loss in CHX207 mice results from increased lipolysis. (A–E) Ten‐ to 11‐week‐old male C57BL/6J mice were injected with 1 × 10^6^ MCA207 cells, 1 × 10^6^ CHX207 cells, or 1× PBS (control). (A) Total fat mass was determined by NMR (*n* = 10–14). (B) Mice were sacrificed with same tumour size and inguinal subcutaneous (iWAT), gonadal (gWAT) white, and interscapular brown adipose tissue (iBAT) were excised and weighed (Day 9 p.i., *n* = 5; Day 13 p.i. and Day 15 p.i., *n* = 8–10; Day 18 p.i., *n* = 3). (C) *In vitro* TG hydrolase activity of gWAT tissue lysates (Day 13 p.i.). (D) *Ex vivo* lipolysis of gWAT fat explants was determined by measuring glycerol release in the presence (+Iso) or absence (basal) of 1 μM isoproterenol (Day 9 p.i., *n* = 9). (E) Western blotting analysis of p‐HSL (Ser660), p‐HSL (Ser565), HSL, CGI‐58 and ATGL in gWAT. VINCULIN was used as loading control (Day 9 p.i.). data are presented as means ± SD. Significance was determined by one‐way ANOVA followed by Tukey's *post hoc* analysis (*P ≤ 0.05, **P ≤ 0.01, ***P ≤ 0.001, ****P ≤ 0.0001).

Using NMR, we found a drastic reduction of fat mass (−49%) in CHX207 mice within 14 days p.i. (Figure 4A), while fat mass did not change in control and MCA207 mice. All adipose tissue depots were significantly reduced 13 days p.i. in CHX207 mice (iWAT: −46%, gWAT: −52%, and iBAT: −45%) compared with MCA207 mice with same tumour weights. Eighteen days p.i., CHX207 mice exhibited an even more severe reduction in iWAT (−66%), gWAT (−73%), and iBAT (−56%) depots compared with control mice (Figure 4B). Adipose tissue weights were also drastically reduced in female CHX207 mice (iWAT: −65%, gWAT: −60%, and iBAT: −36%), compared with female MCA207 mice (Figure S4A).

Adipose tissue wasting is a result of either increased hydrolysis or reduced synthesis of TGs. We found significantly reduced expression of adipo‐lipogenic marker genes, such as FA translocase (*Cd36*, −89%), lipoprotein lipase (*Lpl*, −62%), peroxisome proliferator‐activated receptor gamma2 (*Pparg2*, −61%), CCAAT−/enhancer‐binding protein alpha (*C/ebp1a*, −50%), sterol regulatory element binding protein 1c (*Srebp1c*, −47%), phosphoenolpyruvate carboxykinase (*Pepck*, −83%), and FA synthase (*Fasn*, −57%) in iWAT of CHX207‐ but not MCA207 mice compared with control mice (Figure S4B). Moreover, LPL protein abundance was drastically reduced (−69%) in iWAT of CHX207 mice compared with control and MCA207 mice (Figure S4C). These results indicate that reduced lipid uptake, lipid synthesis, and adipogenesis contribute to a decline of fat mass in CHX207 mice.

Next, we investigated whether increased degradation of TG stores also contributes to adipose tissue loss in CHX207 mice. In accordance with increased *in vitro* TG hydrolase activity (1.7‐fold) in gWAT lysates, gWAT explants of CHX207 mice released 1.6‐fold more glycerol compared with explants of MCA207‐ and control mice *ex vivo* (Figure 4C,D). Beta‐adrenergic stimulation using isoproterenol increased glycerol release further and to 3.7‐fold higher levels in CHX207 gWAT explants compared with MCA207 gWAT explants (Figure 4D). Adipose triglyceride lipase (ATGL) and hormone‐sensitive lipase (HSL) are the two major TG hydrolases in adipose tissue.^18^ Consistent with increased lipolysis, we detected a significant increase in ATGL protein expression in gWAT of CHX207 mice (6‐fold) compared with MCA207‐ or control mice. In addition, protein levels of the ATGL‐coactivator comparative gene identification‐58 (CGI‐58) were drastically increased in gWAT of CHX207 mice (12‐fold). However, neither total HSL protein abundance nor phosphorylation of HSL at Ser660 (which activates the enzyme) or Ser565 (which inhibits enzyme activity) were significantly different in gWAT of all groups (Figure 4E).

To investigate a potential causal role of ATGL in CHX207‐induced cachexia, we assessed CAC progression in mice lacking ATGL in all tissues except for cardiac muscle (AKO) and compared them with tumour‐bearing or non‐tumour‐bearing wild‐type (WT) animals. Within 18 days and with similar tumour weights, WT‐CHX207 and AKO‐CHX207 mice lost 4.4 g and 2.5 g body weight, respectively, compared with control mice (Figure S4D,E). Adipose tissue depots and skeletal muscles were reduced by 56% (iWAT), 80% (gWAT), and 13% (m.qu.) in WT‐CHX207 compared with control mice. Adipose tissue weights were significantly increased in AKO mice compared with WT mice and completely preserved in the presence of the tumour in AKO‐CHX207 mice (Figure S4F). In contrast, ATGL deficiency did not protect AKO‐CHX207 mice from skeletal muscle loss (Figure S4F). This observation indicates that the partial protection from body weight loss in AKO‐CHX207 mice is predominantly due to a complete protection from adipose tissue loss.

Despite increased lipolysis and adipose tissue loss, we did not find differences in circulating glycerol and FAs between MCA207‐, CHX207‐, and control mice (Table S1), indicating that *in vivo* (i) lipolytic products are not released into the circulation but oxidized within adipose tissue and/or (ii) the increasingly liberated metabolites are efficiently taken up by oxidative tissues such as skeletal muscle or liver.

**Figure 5:**
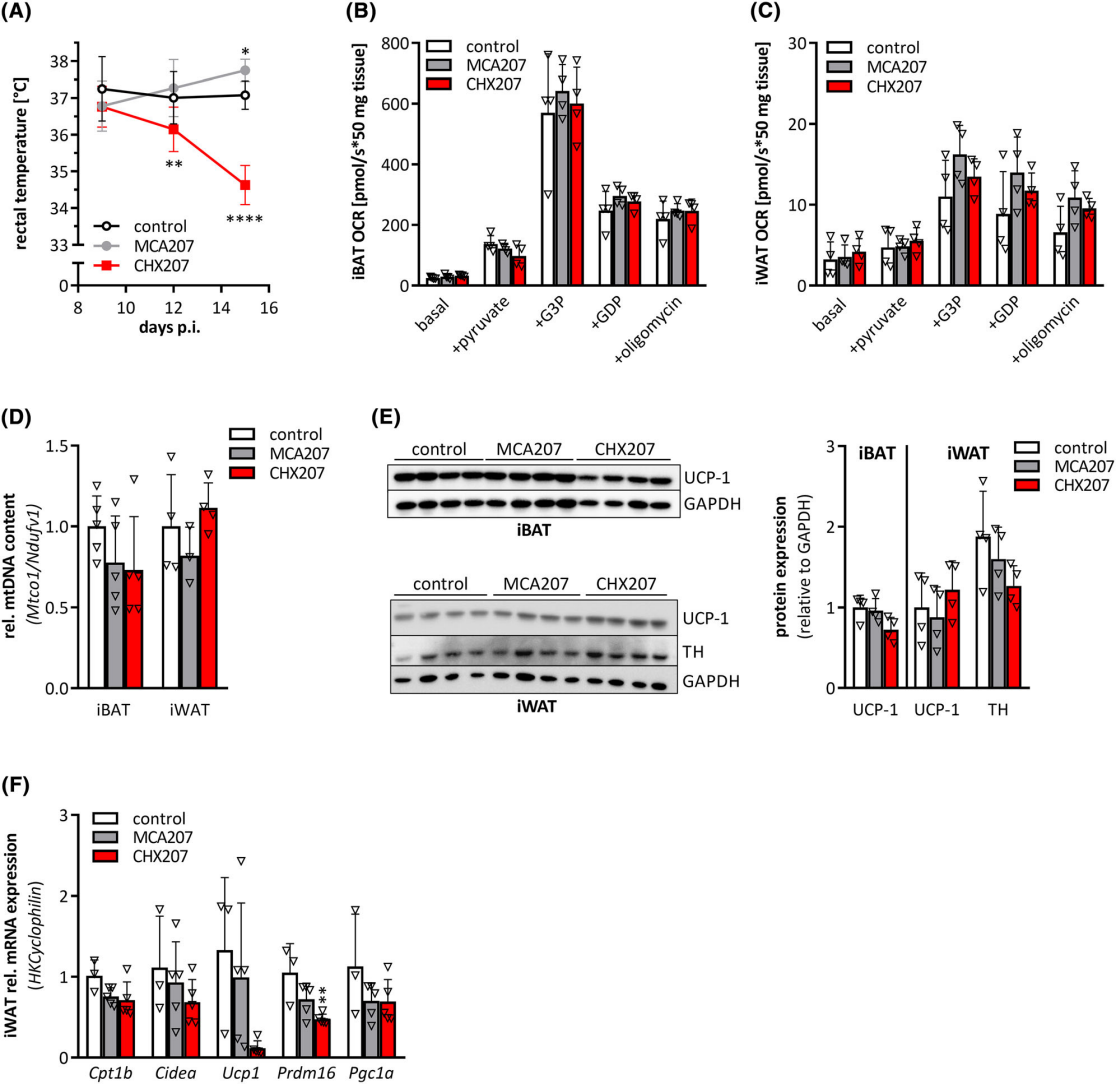
CHX207‐induced cachexia is not associated with browning of WAT. (A–F) Ten‐ to 11‐week‐old male C57BL/6J mice were injected with 1 × 10^6^ MCA207 cells, 1 × 10^6^ CHX207 cells, or 1× PBS (control). (A) Rectal temperature was measured using a rectal probe (*n* = 10–14). (B,C) Oxygen consumption rates (OCRs) of total homogenates of (B) interscapular brown (iBAT), and (C) inguinal subcutaneous (iWAT) white adipose tissue were determined in the presence of pyruvate, glycerol‐3‐phosphate (G3P), guanosine diphosphate (GDP), and oligomycin (oligo) using an oxygraph on Day 9 p.i. (D) Relative mitochondrial content was determined by calculating the ratio of *Mtco1* (mitochondria encoded) and *Ndufv1* (nucleus encoded) mRNA levels of iBAT (*n* = 5) and iWAT (*n* = 4) (Day 13 p.i.). (E) Uncoupling protein‐1 (UCP‐1) protein in iBAT and UCP‐1 and tyrosine hydroxylase (TH) protein levels in iWAT (Day 13 p.i.) were determined by Western blotting analysis. GAPDH was used as loading control. (F) mRNA expression levels of *Cpt1b*, *Cidea*, *Ucp1*, *Prdm16*, and *Pgc1a* in iWAT were determined by qRT‐PCR (Day 9 p.i.). *Cyclophilin* was used as housekeeping gene. Data are presented as means ± SD. Significance was determined by one‐way ANOVA followed by Tukey's *post hoc* analysis (*n* = 4–5, *P ≤ 0.05, **P ≤ 0.01, ***P ≤ 0.001, ****P ≤ 0.0001).

We first delineated whether increased substrate oxidation and energy dissipation by uncoupling protein‐1 (UCP‐1) in BAT and iWAT contribute to adipose tissue loss in CHX207 mice. Body temperature was similar or even decreased in CHX207 mice compared with MCA207 mice (Figure 5A) and oxygen consumption rates were comparable in iBAT and iWAT of all groups (Figure 5B,C). Moreover, mitochondrial DNA content (Figure 5D), UCP‐1 protein expression (Figure 5E) and mRNA levels of thermogenic marker genes such as carnitine palmitoyl transferase 1 beta (*Cpt1b*), cell death inducing DFFA like effector A (*Cidea*), peroxisome proliferator‐activated receptor co‐activator‐1 alpha (*Pgc1a*), *Ucp1*, and PR‐ domain containing protein 16 (*Prdm16*) were unchanged or even reduced in iWAT of CHX207‐ and MCA207‐ compared with control mice (Figure 5F). Finally, protein expression of tyrosine hydroxylase (TH), the rate‐limiting enzyme in catecholamine synthesis, was comparable in iWAT of CHX207‐, MCA207‐, and control mice (Figure 5E). These results exclude a significant induction of UCP‐1 dependent thermogenesis via beta‐adrenergic signalling as a major cause for WAT wasting in CHX207 mice.

To investigate whether CHX207 mice show increased FA clearance from plasma, we performed *in vivo* radioactive tracer studies using ^14^C‐bromo‐palmitic acid (B‐PA). Two minutes after intravenous injection, plasma B‐PA concentrations were similar in all groups (Figure S4G). After additional 15 min, plasma B‐PA concentrations dropped by 60% in control mice, 54% in MCA207 mice, and 70% in CHX207 mice (Figure S4G), indicating a more efficient FA clearance in cachectic mice. An elevated FA flux to the liver has been suggested to contribute to hepatic steatosis and energy deduction in CAC.^2^ We did not observe differences in B‐PA uptake into livers of either control, MCA207‐ or CHX207 mice (Figure S4H) and liver acylglycerol content was reduced by 17% and 46% in MCA207‐ and CHX207 mice, compared with control mice, respectively (Figure S4I). B‐PA uptake was similar in iWAT, gWAT, iBAT, c.m., and m.qu. of all groups (Figure S4H). However, we detected considerably higher B‐PA uptake into tumours of CHX207‐ (2.6‐fold) than of MCA207 mice (Figure S4H). Consistent with elevated lipid utilization for energy conversion in CHX207‐ compared with MCA207 mice, metabolic cage analyses revealed a significantly reduced respiratory exchange ratio (RER; VCO_2_/VO_2_) on Days 12 and 13 p.i. (Figure S4J). This suggests that a combination of reduced adipo‐lipogenesis, increased lipolysis, and increased FA utilization by the tumour contributes to WAT loss in CHX207‐induced cachexia.

### Cancer cell derived IL‐6 triggers cachexia in CHX207 mice

**Figure 6:**
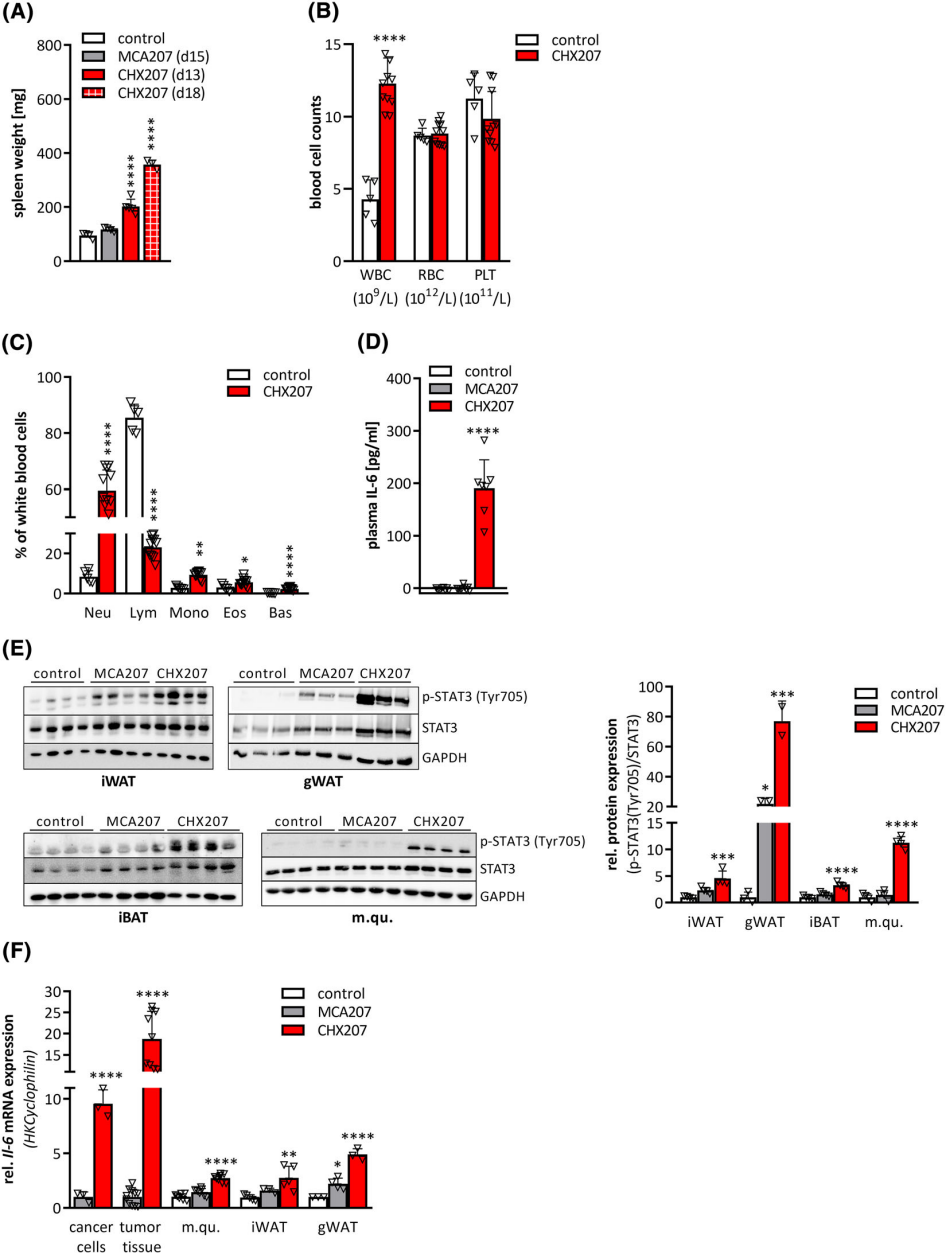
CHX207 mice exhibit systemic inflammation and high levels of circulating IL‐6. (A–F) Ten‐ to 11‐week‐old male C57BL/6J mice were injected with 1 × 10^6^ MCA207 cells, 1 × 10^6^ CHX207 cells, or 1× PBS (control). (A) Mice were sacrificed at the indicated timepoints, spleens were excised and weighed. (*B*,C) Cell counts of whole blood from control and tumour‐bearing mice were analysed using an abacus Haematology analyser (*n* = 5–11, Day 14 p.i.). (B) Absolute counts of white blood cells (WBC), red blood cells (RBC), and platelets (PLT). (C) Relative white blood cells subtypes (neutrophils (Neu), lymphocytes (Lym), monocytes (Mono), eosinophils (Eos), basophils (bas)). (D) Plasma IL‐6 concentrations were determined using ELISA (*n* = 7–9). (E) Western blotting analysis of p‐STAT3 (Tyr705) and STAT3 protein expression and quantification of p‐STAT3 (Tyr705) relative to total STAT3 in inguinal white adipose tissue (iWAT), gonadal white adipose tissue (gWAT), interscapular brown adipose tissue (iBAT), and musculus quadriceps (m.qu.) (Day 13 p.i.). GAPDH was used as loading control. (F) mRNA expression levels of *Il‐6* in MCA207‐ and CHX207‐cancer cells (*n* = 3–4) and tumour tissue, m.qu., iWAT and gWAT (Day 13 p.i., *n* = 7–11) were determined by qRT‐PCR. *Cyclophilin* was used as housekeeping gene. Data are presented as means + SD. Significance was determined by B,C,F) two‐sided Student's *t*‐test or A,D,E,F) one‐way ANOVA (*P ≤ 0.05, **P ≤ 0.01, ***P ≤ 0.001, ****P ≤ 0.0001).

Besides metabolic changes also immunologic alterations cause cancer progression and contribute to CAC.^19^ Increased spleen weights indicated splenic hyperfunction in CHX207 mice (Figure 6A). Blood cell analyses revealed increased white blood cell counts (Figure 6B) due to an absolute and relative increase in neutrophils, basophils, and eosinophils in CHX207 mice (Day 14 p.i.) compared with control mice (Figures 6C and S5A). In contrast, absolute and relative lymphocyte numbers were unchanged or decreased, respectively (Figures 6C and S5A), causing an elevation of the neutrophil to lymphocyte ratio (NLR) from 0.1 to 2.8 (Figure S5B). Although red blood cells and platelets were not different between groups (Figure 6B), monocytes were increased in CHX207 mice compared with control mice (Figures 6C and S5A). These results strongly argued for systemic inflammation in CHX207 mice.

ELISA experiments to quantify circulating cachexokines revealed similar concentrations of leukaemia inhibitory factor (LIF) and myostatin, but 4‐fold elevated PTHrP concentrations in the blood of CHX207‐, compared with MCA207‐ and control mice (Table S1). Concentrations of meteorin‐like glial cell differentiation regulator (METRNL) and TNFα were below the detection limit in all groups. IL‐6 was not detectable in control‐ and MCA207 mice, but reached 190 ± 54 pg/mL in the plasma of CHX207 mice (Day 13 p.i.) (Figure 6D). Elevated systemic IL‐6 concentrations translated into activated IL‐6‐signalling, which was evident by increased phosphorylation of signal transducer and activator of transcription 3 (p‐STAT3) in iWAT (2‐fold), gWAT (3.5‐fold), iBAT (2‐fold), and m.qu. (7.9‐fold), of CHX207‐ compared with MCA207 mice (Figure 6E). Increased circulating IL‐6 levels resulted from elevated *Il‐6* mRNA expression in cancer cells (9.5‐fold), tumour tissue (19‐fold), m.qu. (2‐fold), iWAT (1.7‐fold), and gWAT (2.2‐fold) of CHX207 mice compared with MCA207 mice (Figure 6F).

**Figure 7:**
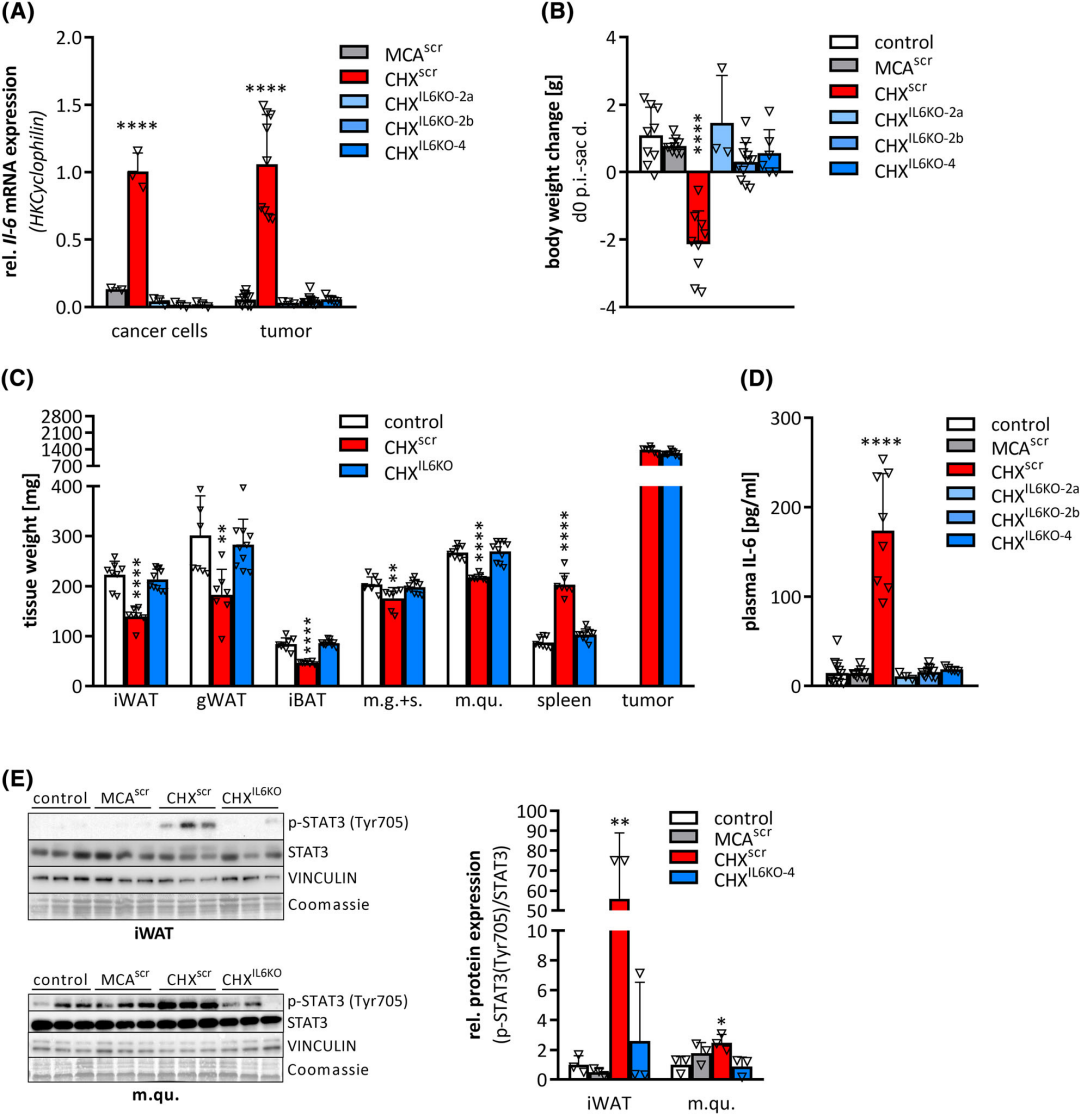
Cancer cell derived IL‐6 initiates cachexia in CHX207 mice. (A–E) Ten‐ to 11‐week‐old male C57BL/6J mice were injected with either 1 × 10^6^ MCA^scr^, CHX^scr^, CHX^IL6KO‐2a^, CHX^IL6KO‐2b^, or CHX^IL6KO‐4^ cells or 1× PBS (control) and were sacrificed with same tumour size (1.4 g) (*n* = 7–11, except for CHX^IL6KO‐2a^ *n* = 3). (A) mRNA expression levels of *Il‐6* in cancer cells and tumour tissue were determined by qRT‐PCR. *Cyclophilin* was used as housekeeping gene. (B) Body weight change from Day 0 p.i. (d0) to day of sacrifice (sac d.) (tumour weight was subtracted). (C) Inguinal subcutaneous (iWAT), gonadal (gWAT) white, interscapular brown adipose tissue (iBAT), musculus gastrocnemius + soleus (m.g. + s.), musculus quadriceps (m.qu.), spleen, and tumour were excised and weighed. (D) Plasma IL‐6 concentrations were determined by ELISA. (E) Western blotting analysis to detect p‐STAT3 (Tyr705) and STAT3 protein expression and quantification of p‐STAT3 (Tyr705) relative to total STAT3 in iWAT and m.qu. VINCULIN and Coomassie stain were used as loading controls. Data are presented as means + SD. Significance was determined by one‐way ANOVA (*P ≤ 0.05, **P ≤ 0.01, ***P ≤ 0.001, ****P ≤ 0.0001).

To investigate whether cancer cell‐derived IL‐6 is crucial for cachexia development, we deleted the *Il‐6* gene from CHX207 cells (CHX^IL6KO^) using CRISPR‐Cas9 mediated gene editing (Figure S6A). Similarly transfected cell‐clones using scrambled guide RNAs were used as controls (CHX^scr^ and MCA^scr^). Successful gene deletion in three different CHX^IL6KO^ cell‐clones was verified by qRT‐PCR analyses of cancer cells and tumour tissues (Figure 7A). Although cell proliferation was similar *in vitro*, CHX^IL6KO^ tumours grew significantly slower than CHX^scr^ tumours *in vivo* (Figures S6B,C). At Day 13 p.i., CHX^scr^ mice exhibited reduced tumour‐free body weight (−22%) (Figure 7B), a prominent reduction in adipose tissue‐ and skeletal muscle weights, marked splenomegaly (Figures 7C), and high plasma IL‐6 concentrations (Figure 7D). In contrast, CHX^IL6KO^ mice had similar body weight, adipose tissues‐, skeletal muscles‐, and spleen weights compared with non‐tumour‐bearing control mice, despite the same tumour burden as CHX^scr^ mice (Figure 7B,C). Moreover, CGI‐58 protein content of adipose tissue was reduced to control levels in CHX^IL6KO^ mice (Figure S6E). Deletion of IL‐6 from cancer cells also reduced *Il‐6* mRNA expression in m.qu., iWAT, and gWAT of CHX^IL6KO^ mice (Figure S6D) and resulted in a drastic reduction of plasma IL‐6 concentrations (−94%) as well as p‐STAT3 (Tyr705) levels in iWAT (−60%) and m.qu. (−40%) of male CHX^IL6KO^ mice compared with male CHX^scr^ mice (Figure 7D,E), suggesting that cancer cell‐derived IL‐6 initiates cachexia in CHX207 mice.

Taken together, with CHX207 fibrosarcoma, we present a novel cancer model for C57BL/6 mice that induces robust and reproducible IL‐6 dependent cachexia. CHX207‐induced cachexia is associated with systemic inflammation and metabolic imbalance favouring catabolism over anabolism that causes progressive loss of adipose tissue and skeletal muscle.

## Discussion

A major reason for the lack of suitable therapeutic options for CAC resides in the limited knowledge of the mechanisms causing CAC. Preclinical models are crucial for our understanding of cachexia pathophysiology and the identification of mechanisms that can be targeted to treat the disease. To date, only a few tumour models exist for studying cachexia in C57BL/6 mice. This is unfortunate because C57BL/6 is the best‐studied mouse model in metabolism research and perfectly suited to investigate the role of metabolic derangements in the pathogenesis of CAC. Here, we present CHX207 fibrosarcoma cells, a subtype of MCA207 cells, which are syngeneic to a C57BL/6 genetic background, as a novel allograft cancer model that robustly induces cachexia. Mice that were inoculated with CHX207 or MCA207 cells grew soft‐tissue tumours consisting of highly proliferating parallelly arranged spindle‐shaped fibroblasts with elongated nuclei, closely resembling human fibrosarcoma.^15^ CHX207‐ but not MCA207‐tumour‐bearing mice developed major characteristics of CAC including body weight loss, systemic inflammation, anorexia, and increased energy expenditure.

Patients with fibrosarcoma, a highly aggressive subtype of soft‐tissue sarcoma, often experience weight loss.^15^ Systemic inflammation and high circulating IL‐6 levels are associated with poor prognosis in patients with soft tissue sarcoma.^20^ ^[^ ^S4^ ^]^ In line, mice bearing CHX207 tumours exhibited systemic inflammation and strikingly high circulating concentrations of IL‐6, which was not observed in MCA207 mice. IL‐6 likely plays a causal role in CAC development because cancer cell‐specific deletion of IL‐6 protected CHX207^IL6‐KO^ mice from cachexia. IL‐6 was previously shown to trigger CAC in different murine cancer models^6^, ^21^, ^22^ and IL‐6 plasma concentrations correlate with poor treatment response and increased mortality in cancer patients.^23^ In CHX207 mice, deletion of IL‐6 exclusively in cancer cells was sufficient to prevent CAC even though host cells such as immune cells, myocytes, and adipocytes also produce high levels of IL‐6 in response to cancer.^24^, ^25^ In CHX207^IL6‐KO^ mice, host tissue IL‐6 expression was completely blunted arguing for the existence of a previously described feed‐forward regulatory loop of host tissue IL‐6 expression in response to IL‐6 production in cancer cells.^21^ IL‐6 activated STAT3‐signalling in skeletal muscles and adipose tissues resulted in tissue atrophy of CHX207 mice, similar to previous observations in C26‐tumour‐bearing mice.^26^, ^27^ In line, depending on the animal model, genetic deletion or pharmacological inhibition of IL‐6 or IL‐6 receptor protected mice to a varying degree from body weight loss by either ameliorating adipose tissue or skeletal muscle wasting.^6^, ^21^, ^28^, ^29^ Clinical studies also reported reduced inflammation and amelioration of body weight loss by IL‐6 signalling pathway inhibition,^30^, ^31^ indicating that targeting IL‐6 signalling may be a promising adjunct strategy for cancer treatment. The crucial dependence of the CHX207 model on IL‐6 renders it suitable for studying the efficacy of STAT3 inhibitors (e.g. sorafenib), IL‐6‐, or IL‐6 receptor blocking antibodies (e.g. tocilizumab) to prevent fibrosarcoma‐induced cachexia.

Previous studies indicated that an adipose‐skeletal muscle signalling axis may exist where adipose tissue loss precedes and triggers skeletal muscle wasting in cachexia.^21^, ^32^, ^33^ The importance of adipose tissue loss in the pathogenesis of CAC is also highlighted by the fact that adipose tissue reduction is associated with poor survival of patients with large B cell lymphoma or pancreatic dual carcinoma even in the absence of skeletal muscle wasting.^34^, ^35^ Focusing on the impact of CHX207 and MCA207 malignancies on changes in adipose tissue metabolism, we found reduced LPL expression, reduced adipogenesis, and increased lipolysis to cause WAT loss in CHX207 mice. Although this finding was not unexpected and confirmed previous studies,^36^ the actual triggers for these metabolic rearrangements are still elusive, but likely involve IL‐6 signalling.^[^ ^S5,S6^ ^]^

Lipolysis in adipose tissue is catalysed by ATGL and HSL, and both enzymes have been shown to significantly contribute to adipose tissue loss in CAC in other mouse models.^6^, ^33^, ^37^ We detected increased ATGL and CGI‐58 but not HSL protein abundance in WAT of CHX207 mice. The induction of ATGL may be causal for adipose tissue loss in CHX207 mice because ATGL deficiency protected CHX207 mice from adipose tissue loss. These results are in accordance with previous findings in LLC‐, B16–,^33^ and burn‐induced cachexia^38^ where the deletion or inhibition of ATGL had a similar protective effect. However, ATGL deficiency did not prevent skeletal muscle wasting in CHX207 mice. This finding differs from other models of cachexia but may be due to very high IL‐6 plasma concentrations in CHX207 mice, which was not observed in B16‐ and LLC‐tumour‐bearing animals.^33^ It has been shown that IL‐6, secreted from cancer cells, directly affects muscle metabolism,^26^, ^27^ which is in line with our finding that CHX207‐conditioned medium caused a reduction in myotube diameter and indicates that fat and muscle catabolism are not causally linked in the CHX207 model of CAC.

Earlier works indicated that adipose tissue atrophy is mainly driven by increased sympathetic nerve activity, beta‐adrenergic stimulation of lipolysis, and energy dissipation in WAT.^6^, ^39^ Divergent from these models, CHX207 mice did not exhibit increased beta‐adrenergic signalling, thermogenic marker gene expression or oxygen consumption rates in adipose tissue. Instead of being oxidized in WAT, the increasingly liberated lipolytic products are more efficiently taken up by CHX207 tumours than MCA207 tumours, which probably accelerates WAT loss in CHX207 mice.

With the CHX207 mouse model of CAC we offer an important addition to the limited set of animal models available for cachexia research in C57BL/6 mice. CHX207 fibrosarcoma robustly induces cachexia in male and female mice and can be directly compared with its parental and genetically related non‐cachexigenic MCA207 fibrosarcoma. CHX207 mice share important features of CAC with other allograft cancer models such as C26, including the IL‐6 dependence of CAC pathogenesis and many aspects of lipid and muscle metabolism. However, the absence of hepatosteatosis, sustained beta‐adrenergic activation, WAT browning, and thermogenesis induction represent distinct differences to other models and suggest that IL‐6 acts via alternative mechanisms to induce CAC in CHX207 mice.

## Funding

This work was supported by the SFB‐Immunometabolism [F83], SFB‐Lipid‐Hydrolysis [F7302], by the European Research Council LipoCheX [340896], the Leducq Foundation [12CVD04], the Louis Jeantet Foundation [Louis Jeantet Prize 2015], DK Molecular Enzymology [W901], Austrian Science Fund (FWF) stand‐alone grant LipoLUNG [P30968], FWF‐joint‐project [I 5618], DOC fellowship [25049] of the Austrian Academy of Science (OEAW), aH2020‐MSCA‐ITN (ITN‐2019‐859860 ‐ CANCERPREV), BioTechMed‐Flagship Project ‘Midas’, the University of Graz, and the Medical University of Vienna.

## Conflicts of interest

The authors of this manuscript declare that they have no conflicts of interest.

## Supporting information




**Figure S1** Characteristics of CHX207 and MCA207 tumours and their effect on body weight in C57BL/6 J mice. A‐H) Ten‐ to 11‐week‐old male and female C57BL/6 J mice were injected with 1x10^6^ MCA207, 1x10^6^ CHX207 cells, or 1xPBS (control). A) Paramagnetic visualization and transversal tissue relaxation rate R2* for visualization of acute tumour hematomas (pre‐cachexia = d7 (*n* = 8), cachexia = d9‐d13 (*n* = 24–27)). B) Representative MR images of tumours featuring heat map analysis of R2* for visualization of acute tumour hematomas (day 9 p.i.). C) Representative images of immunohistochemical analyses of CD31 protein abundance in tumour sections (scale bar 200 μm, day 9 p.i.). D) Representative images of H&E‐stained lung whole‐cross sections for metastasis analysis (scale bar 200 μm, day 13 p.i.). E) Transcriptomic analysis of MCA207 and CHX207 tumours excised at day 9 p.i. and day 12 p.i. (*n* = 5). F) Body weight relative to initial body weight of female control‐ and tumour‐bearing mice (*n* = 4–5). G) Tumour weight, and H) tumour‐free body weight of female mice (n = 4–5, day 15 p.i. (d15)). I) Correlation plot of body weight and tumour size. Data are presented as means ± s.d. Significance was determined by A,E,G,I) two‐sided Student's t‐test, or F,H) one‐way ANOVA followed by Tukey's *post hoc* analysis (*p ≤ 0.05, **p ≤ 0.01, ***p ≤ 0.001, ****p ≤ 0.0001).
**Figure S2** Systemic metabolic changes in CHX207‐ compared to MCA207 mice. A‐D) Ten‐ to 11‐week‐old male C57BL/6 J mice were injected with 1x10^6^ MCA207 cells, 1x10^6^ CHX207 cells, or 1xPBS (control). A) Activity and energy expenditure were determined using a laboratory animal monitoring system (Phenomaster, TSE Systems GmbH). B) Energy expenditure at equal activity (1834 bb/h) was determined using analysis of covariance (ANCOVA). C) Targeted metabolomics to determine free carnitine, total phosphatidylcholine (PC) and total sphingomyelins in plasma of CHX207 (day 18 p.i.) and MCA207 (day 22 p.i.) mice were performed using the AbsoluteIDQ®p180 assay kit (Biokrates). D) Heat map presenting differences in plasma sphingomyelins species of CHX207‐ and MCA207 mice (data are presented as fold change relative to mean of MCA207 mice). Data are presented as means + s.d. Significance was determined by A) one‐way ANOVA followed by Tukey's *post hoc* analysis, or B) ANCOVA analysis, or C‐D) two‐sided Student's t‐test (*n* = 4–5, *p ≤ 0.05, **p ≤ 0.01, ***p ≤ 0.001).
**Figure S3** CHX207‐induced skeletal muscle wasting results from increased catabolic signalling. A‐E) Ten‐ to 11‐week‐old male C57BL/6 J mice were injected with 1x10^6^ MCA207 cells, 1x10^6^ CHX207 cells, or 1xPBS (control). A) Relative difference of muscle weights [musculus gastrocnemius soleus (m.g. + s.), musculus quadriceps (m.qu.) and cardiac muscle (c.m.)] of male and female control and tumour‐bearing mice (day 13/15 p.i., *n* = 6–10, ‘*’ control vs. CHX207, ‘#’ male vs. female). B) Total acylglycerol of m.qu. (day 13 p.i., *n* = 5–9) was measured using Infinity Triglycerides Reagent. C) Plasma creatine kinase (CK) activity was measured using N‐Acetyl Cysteine (CK‐NAC) reagent (day 18 p.i.). C‐D) Western blotting analysis and signal quantification of m.qu. of control and tumour‐bearing mice (day 13 p.i.). VINCULIN was used as loading control. D) K48‐linkage specific polyubiquitinylation and LC3B expression. E) CASPASE 3 expression. Data are presented as means + s.d. Significance was determined by one‐way ANOVA followed by Tukey's *post hoc* analysis (*n* = 3–5, *p ≤ 0.05, **p ≤ 0.01, ***p ≤ 0.001; ****p ≤ 0.0001).
**Figure S4** CHX207 mice exhibit decreased adipolipogenesis, increased FA uptake into tumours, and ATGL deficient mice (AKO/cTg) are protected from CHX207‐induced adipose tissue loss. A‐C, G‐J) Ten‐ to 11‐week‐old C57BL/6 J mice were injected with 1x10^6^ MCA207 cells, 1x10^6^ CHX207 cells, or 1xPBS (control) and were sacrificed with same tumour size (day 15 p.i. for females, day 13 or 15 p.i. for males). A) Inguinal subcutaneous (iWAT), gonadal (gWAT) white, and interscapular brown adipose tissue (iBAT) of female C57BL/6 J mice were excised and weighed. B) mRNA expression levels of marker genes for lipid uptake (*Cd36*, *Lpl*) and adipogenesis/lipogenesis (*Pparg2*, *C/ebp1a*, *Srebp1c*, *Pepck*, *Fasn*, *Dgat2*) in iWAT were determined by qRT‐PCR. *Cyclophilin* was used as housekeeping gene (male, day 13 p.i.). C) LPL protein expression in iWAT was determined by Western blotting analysis. VINCULIN was used as loading control (male, day 13 p.i.). D‐F) Fifteen week old male and female wildtype (WT) and ATGL‐ko (AKO) mice were injected with 1x10^6^ CHX207 cells or 1xPBS (control) and sacrificed on day 18 p.i. D) Tumour weight (*n* = 5), E) body weight change (tumour weight was subtracted) within 18 days (*n* = 6–7), and F) weights of iWAT, gWAT, iBAT, and musculus quadriceps (m.qu.) were determined (n = 6–10). G‐H) ^14^C labelled bromo‐palmitic acid (B‐PA, 1 μCi/mouse, *n* = 4, day 13 p.i.) was intravenously injected and levels of B‐PA were measured by liquid scintillation counting in plasma, iWAT, gWAT, iBAT, cardiac muscle (c.m.), musculus quadriceps (m.qu.), livers and tumours. I) Total acylglycerol of livers (day 13 p.i., *n* = 12–13) was measured using Infinity Triglycerides Reagent. J) Respiratory exchange ratio (RER; VCO_2_/VO_2_) was measured in a laboratory animal monitoring system (*n* = 4, day 12–13 p.i., light and dark phase). Data are presented as means + s.d. Significance was determined by A‐C,E‐J) one‐way ANOVA followed by Tukey's *post hoc* or D) two‐sided Student's t‐test analysis (*n* = 3–5,* *p* ≤ 0.05, **p ≤ 0.01, ***p ≤ 0.001, ****p ≤ 0.0001).
**Figure S5** CHX207 mice exhibit altered blood counts. A‐B) Ten‐ to 11‐week‐old male C57BL/6 J mice were injected with 1x10^6^ CHX207 cells, or 1xPBS (control). Cell counts of whole blood from control and tumour‐bearing mice were analysed using an Abacus Haematology analyser (*n* = 5–11, day 14 p.i.). A) Absolute counts of white blood cells subtypes (Neutrophils (Neu), Lymphocytes (Lym), Monocytes (Mono), Eosinophils (Eos), Basophils (Bas)). B) Neutrophil to Lymphocyte Ratio (NLR) of absolute cell counts. Data are presented as means + s.d.. Significance was determined by two‐sided Student's t‐test (*p ≤ 0.05, **p ≤ 0.01, ***p ≤ 0.001, ****p ≤ 0.0001).
**Figure S6** Crispr‐Cas9‐mediated *Il‐6* silencing slows tumour growth and reduces IL‐6 signalling in CHX207 mice. A) (1) Scheme of the murine *Il‐6* gene (NM 031168). (2) Appropriate gRNAs targeting exon 2 (^IL6KO‐2a^, ^IL6KO‐2b^) and exon 4 (^IL6KO‐4^) of *Il‐6* for *S. pyogenes* Cas9 were designed. gRNAs were designed including a 5 overhang for ligation into BbsI sites (5‐CACCG) of the plasmid pSpCas9 (BB) (Addgene #62988). (3) T4 PNK‐phosphorylated gRNAs were ligated into BbsI‐digested plasmids pSpCas9(BB)‐2A‐Puro (PX459) V2.0 via Qick Ligase (NEB #M2200). (4) Plasmid‐gRNA‐constructs were transfected into MCA207 and CHX207 cells via Turbofect™ (Thermo Fisher #R0534). (5) Transfected MCA207 and CHX207 cells were selected with 5 μM puromycin. Puromycin‐resistant cells were diluted to single cells and clonal expansion of cells was performed. *Il‐6*‐knock out of cells was verified by qRT‐PCR and 3 different CHX^IL6KO^ cell lines, MCA^scr^ and CHX^scr^ cells were injected into C57BL/6 J mice. B) Cell proliferation of CHX^scr^, CHX^IL6KO‐2a^, CHX^IL6KO‐2b^ and CHX^IL6KO‐4^ cells in culture was determined by counting the cells for 7 consecutive days after seeding (*n* = 3). C‐E) Ten‐ to 11‐week‐old male C57BL/6 J mice were injected with either 1x10^6^ MCAscr, CHXscr, MCA207, CHX207, CHX^IL6KO‐2a^, CHX^IL6KO‐2b^ or CHX^IL6KO‐4^ cells or 1xPBS (control) and sacrificed with same tumour size (1.4 g). C) Tumour diameters from day 8 p.i. until day of sacrifice were measured using a sliding calliper and linear regression analysis of tumour growth was performed. D) mRNA expression levels of *Il‐6* in musculus quadriceps (m.qu.), inguinal white (iWAT), and gonadal white adipose tissue (gWAT) were determined by qRT‐PCR. *Cyclophilin* was used as housekeeping gene (*n* = 3–7, day 13 p.i.). E) Western blotting analysis and quantification of CGI‐58 protein expression in iWAT and gWAT of control and tumour‐bearing mice (day 9 p.i.). VINCULIN was used as loading control. Data are presented as means ± s.d. Significance was determined by one‐way ANOVA (n = 3–11, *p ≤ 0.05, **p ≤ 0.01, ***p ≤ 0.001).

**Table S1.** Plasma parameters and cytokine levels of control mice, MCA207 mice, and CHX207 mice. Data are presented as means ± s.d. Significance was determined by two‐sided Student's t‐test (Amino acids) or one‐way ANOVA followed by Tukey's post hoc analysis (*n* = 4–17, * p ≤ 0.05, **p ≤ 0.01, ***p ≤ 0.001, ****p ≤ 0.0001, n.d. = not detected).

**Table S2.** FPKM of tumor transcriptomic analyses

**Data S1.** Supplemental References

**Data S2.** Supplemental Methods
