# Supplementary material for: Interleukin‐6 initiates muscle‐ and adipose tissue wasting in a novel C57BL/6 model of cancer‐associated cachexia
Source: J Cachexia Sarcopenia Muscle. 2022 Nov 9;14(1):93–107. doi: 10.1002/jcsm.13109 (PMC9891934; doi:10.1002/jcsm.13109)
Supplement: Supplementary file 1 — Figure S1 Characteristics of CHX207 and MCA207 tumours and their effect on body weight in C57BL/6 J mice. A‐H) Ten‐ to 11‐week‐old male and female C57BL/6 J mice were injected with 1x10^6^ MCA207, 1x10^6^ CHX207 cells, or 1xPBS (control). A) Paramagnetic visualization and transversal tissue relaxation rate R2* for visualization of acute tumour hematomas (pre‐cachexia = d7 (n = 8), cachexia = d9‐d13 (n = 24–27)). B) Representative MR images of tumours featuring heat map analysis of R2* for visualization of acute tumour hematomas (day 9 p.i.). C) Representative images of immunohistochemical analyses of CD31 protein abundance in tumour sections (scale bar 200 μm, day 9 p.i.). D) Representative images of H&E‐stained lung whole‐cross sections for metastasis analysis (scale bar 200 μm, day 13 p.i.). E) Transcriptomic analysis of MCA207 and CHX207 tumours excised at day 9 p.i. and day 12 p.i. (n = 5). F) Body weight relative to initial body weight of female control‐ and tumour‐bearing mice (n = 4–5). G) Tumour weight, and H) tumour‐free body weight of female mice (n = 4–5, day 15 p.i. (d15)). I) Correlation plot of body weight and tumour size. Data are presented as means ± s.d. Significance was determined by A,E,G,I) two‐sided Student's t‐test, or F,H) one‐way ANOVA followed by Tukey's post hoc analysis (*p ≤ 0.05, **p ≤ 0.01, ***p ≤ 0.001, ****p ≤ 0.0001). Figure S2 Systemic metabolic changes in CHX207‐ compared to MCA207 mice. A‐D) Ten‐ to 11‐week‐old male C57BL/6 J mice were injected with 1x10^6^ MCA207 cells, 1x10^6^ CHX207 cells, or 1xPBS (control). A) Activity and energy expenditure were determined using a laboratory animal monitoring system (Phenomaster, TSE Systems GmbH). B) Energy expenditure at equal activity (1834 bb/h) was determined using analysis of covariance (ANCOVA). C) Targeted metabolomics to determine free carnitine, total phosphatidylcholine (PC) and total sphingomyelins in plasma of CHX207 (day 18 p.i.) and MCA207 (day 22 p.i.) mice were performed using the A [file JCSM-14-93-s005.docx]

**Supplementary Figures and Legends**

**Interleukin-6 initiates muscle- and adipose tissue wasting in a novel C57BL/6 model of cancer-associated cachexia**

*Journal of Cachexia, Sarcopenia and Muscle*

Isabella Pototschnig, Ursula Feiler, Clemens Diwoky, Paul W. Vesely, Thomas Rauchenwald, Margret Paar, Latifa Bakiri, Laura Pajed, Peter Hofer, Karl Kashofer, Nyamdelger Sukhbaatar, Gabriele Schoiswohl, Thomas Weichhart, Gerald Hoefler, Christoph Bock, Martin Pichler, Erwin F. Wagner, Rudolf Zechner, and Martina Schweiger^#^

# Corresponding author affiliation: Institute of Molecular Biosciences, University of Graz, Graz, Austria

Email: [tina.schweiger@uni-graz.at](mailto:tina.schweiger@uni-graz.at)

**
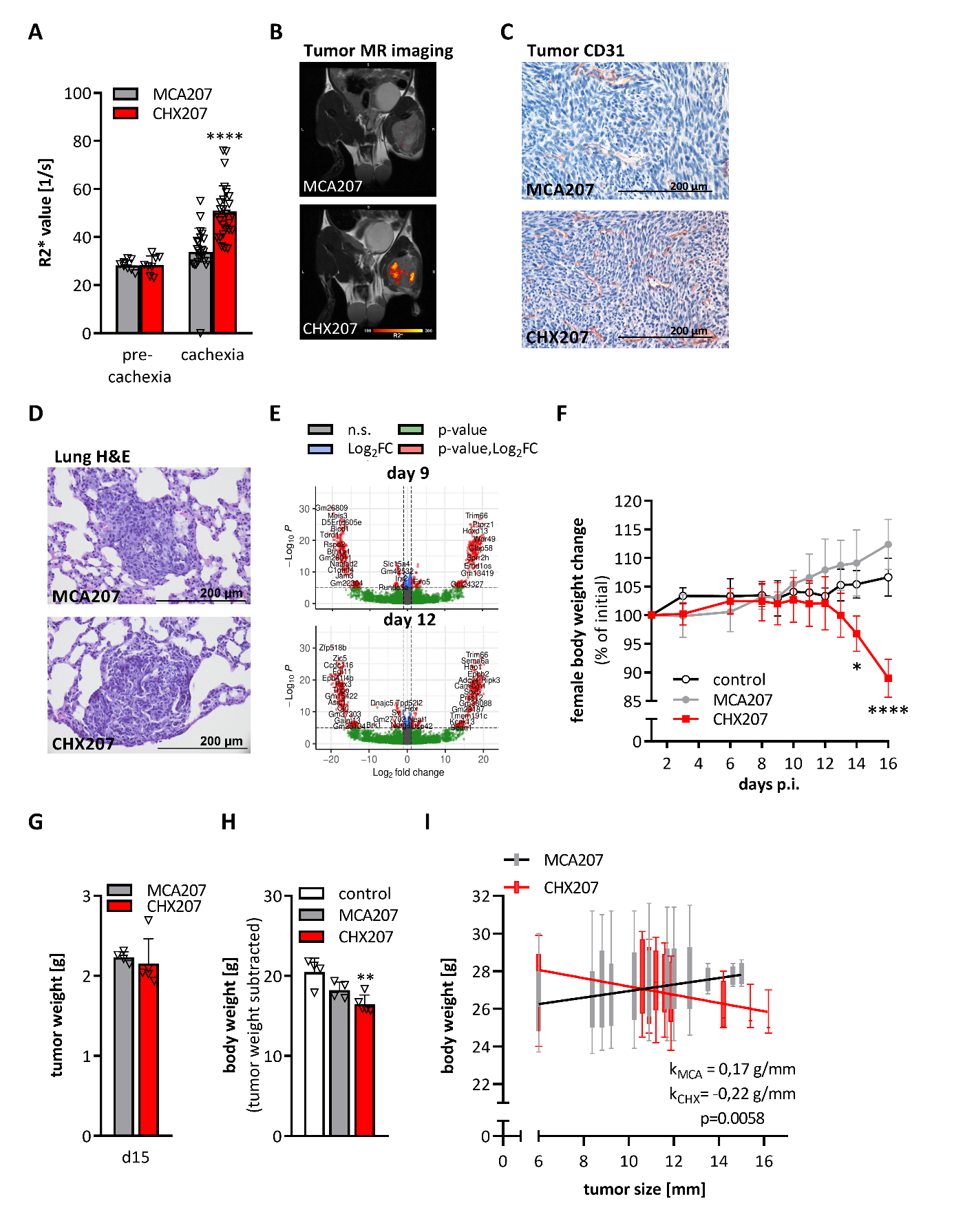
**

**Figure S1** Characteristics of CHX207 and MCA207 tumors and their effect on body weight in C57BL/6J mice. **A-H)** Ten- to 11-week-old male and female C57BL/6J mice were injected with 1x10^6^ MCA207, 1x10^6^ CHX207 cells, or 1xPBS (control). **A)** Paramagnetic visualization and transversal tissue relaxation rate R2* for visualization of acute tumor hematomas (pre-cachexia=d7 (n=8), cachexia=d9-d13 (n=24-27)). **B)** Representative MR images of tumors featuring heat map analysis of R2* for visualization of acute tumor hematomas (day 9 p.i.). **C)** Representative images of immunohistochemical analyses of CD31 protein abundance in tumor sections (scale bar 200 µm, day 9 p.i.). **D)** Representative images of H&E-stained lung whole-cross sections for metastasis analysis (scale bar 200 µm, day 13 p.i.). **E)** Transcriptomic analysis of MCA207 and CHX207 tumors excised at day 9 p.i. and day 12 p.i. (n=5). **F)** Body weight relative to initial body weight of female control- and tumor-bearing mice (n=4-5). **G)** Tumor weight, and **H)** tumor-free body weight of female mice (n=4-5, day 15 p.i. (d15)). **I)** Correlation plot of body weight and tumor size. Data are presented as means ± s.d.. Significance was determined by **A,E,G,I)** two-sided Student’s t-test, or **F,H)** one-way ANOVA followed by Tukey’s *post hoc* analysis (*p≤0.05, **p ≤ 0.01, ***p ≤ 0.001, ****p ≤ 0.0001).

**
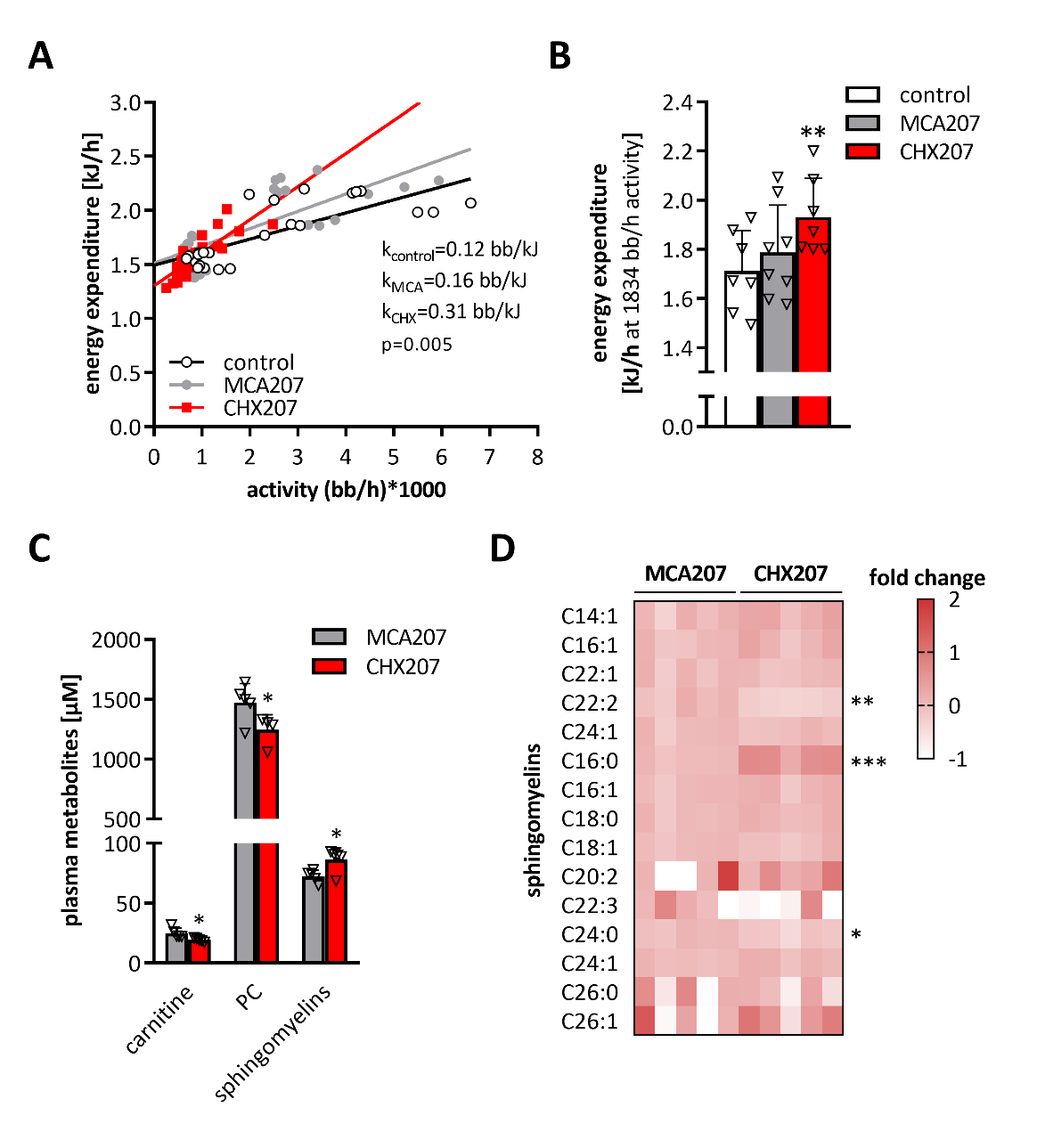
**

**Figure S2** Systemic metabolic changes in CHX207- compared to MCA207 mice. **A-D)** Ten- to 11-week-old male C57BL/6J mice were injected with 1x10^6^ MCA207 cells, 1x10^6^ CHX207 cells, or 1xPBS (control). **A)** Activity and energy expenditure were determined using a laboratory animal monitoring system (Phenomaster, TSE Systems GmbH). **B)** Energy expenditure at equal activity (1834 bb/h) was determined using analysis of covariance (ANCOVA)**. C)** Targeted metabolomics to determine free carnitine, total phosphatidylcholine (PC) and total sphingomyelins in plasma of CHX207 (day 18 p.i.) and MCA207 (day 22 p.i.) mice were performed using the AbsoluteIDQ®p180 assay kit (Biokrates). **D)** Heat map presenting differences in plasma sphingomyelins species of CHX207- and MCA207 mice (data are presented as fold change relative to mean of MCA207 mice). Data are presented as means + s.d.. Significance was determined by **A)** one-way ANOVA followed by Tukey’s *post hoc* analysis, or **B)** ANCOVA analysis, or **C-D)** two-sided Student’s t-test (n=4-5, *p≤0.05, **p ≤ 0.01, ***p ≤ 0.001).


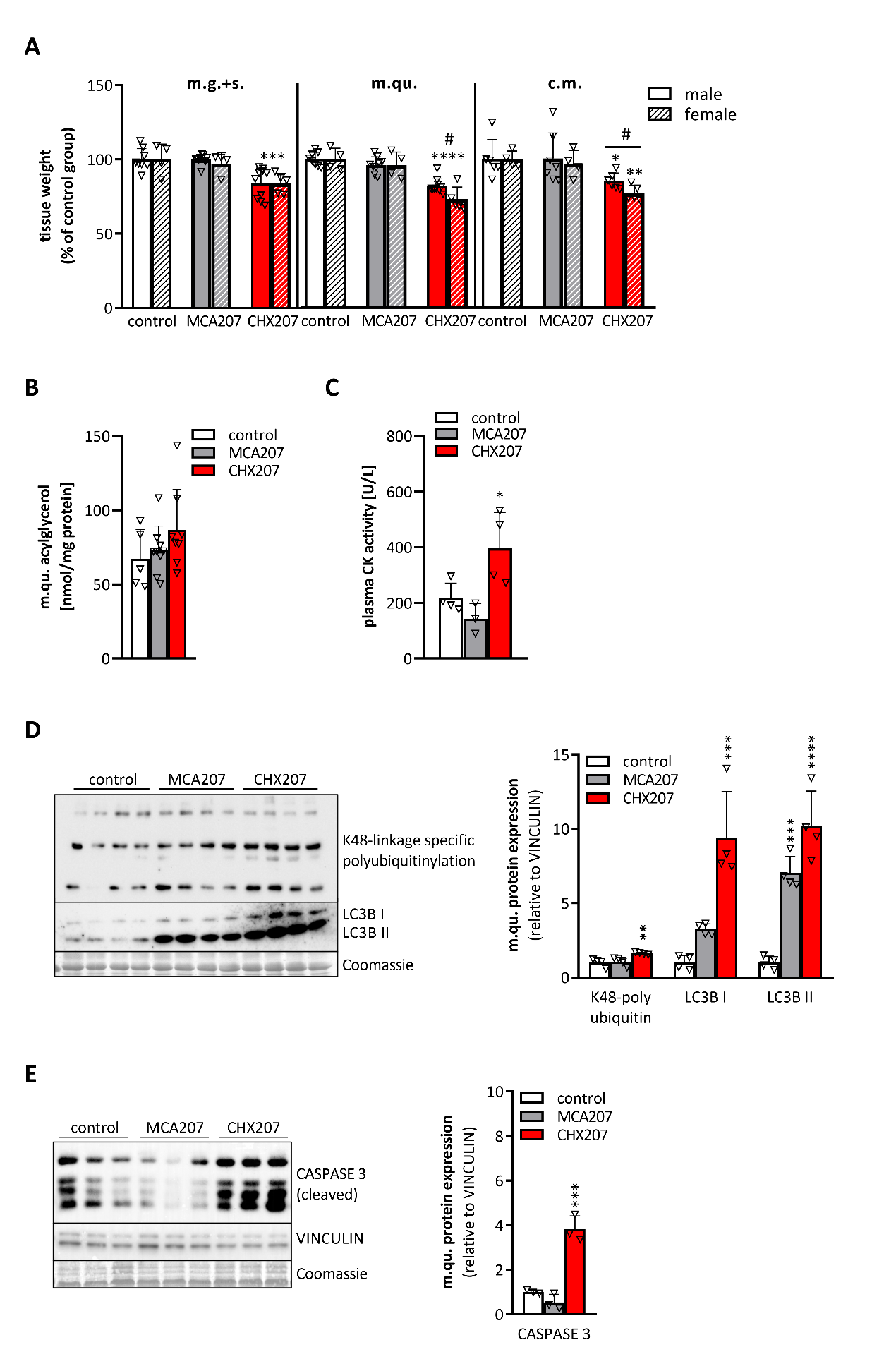


**Figure S3** CHX207-induced skeletal muscle wasting results from increased catabolic signaling. **A-E)** Ten- to 11-week-old male C57BL/6J mice were injected with 1x10^6^ MCA207 cells, 1x10^6^ CHX207 cells, or 1xPBS (control). **A)** Relative difference of muscle weights [musculus gastrocnemius soleus (m.g.+s.), musculus quadriceps (m.qu.) and cardiac muscle (c.m.)] of male and female control and tumor-bearing mice (day 13/15 p.i., n=6-10, '*' control vs. CHX207, '#' male vs. female). **B)** Total acylglycerol of m.qu. (day 13 p.i., n=5-9) was measured using Infinity Triglycerides Reagent. **C)** Plasma creatine kinase (CK) activity was measured using N-Acetyl Cysteine (CK-NAC) reagent (day 18 p.i.). C-D) Western blotting analysis and signal quantification of m.qu. of control and tumor-bearing mice (day 13 p.i.). VINCULIN was used as loading control. **D)** K48-linkage specific polyubiquitinylation and LC3B expression. **E)** CASPASE 3 expression. Data are presented as means + s.d.. Significance was determined by one-way ANOVA followed by Tukey’s *post hoc* analysis (n=3-5, *p≤0.05, **p ≤ 0.01, ***p ≤ 0.001; ****p ≤ 0.0001).


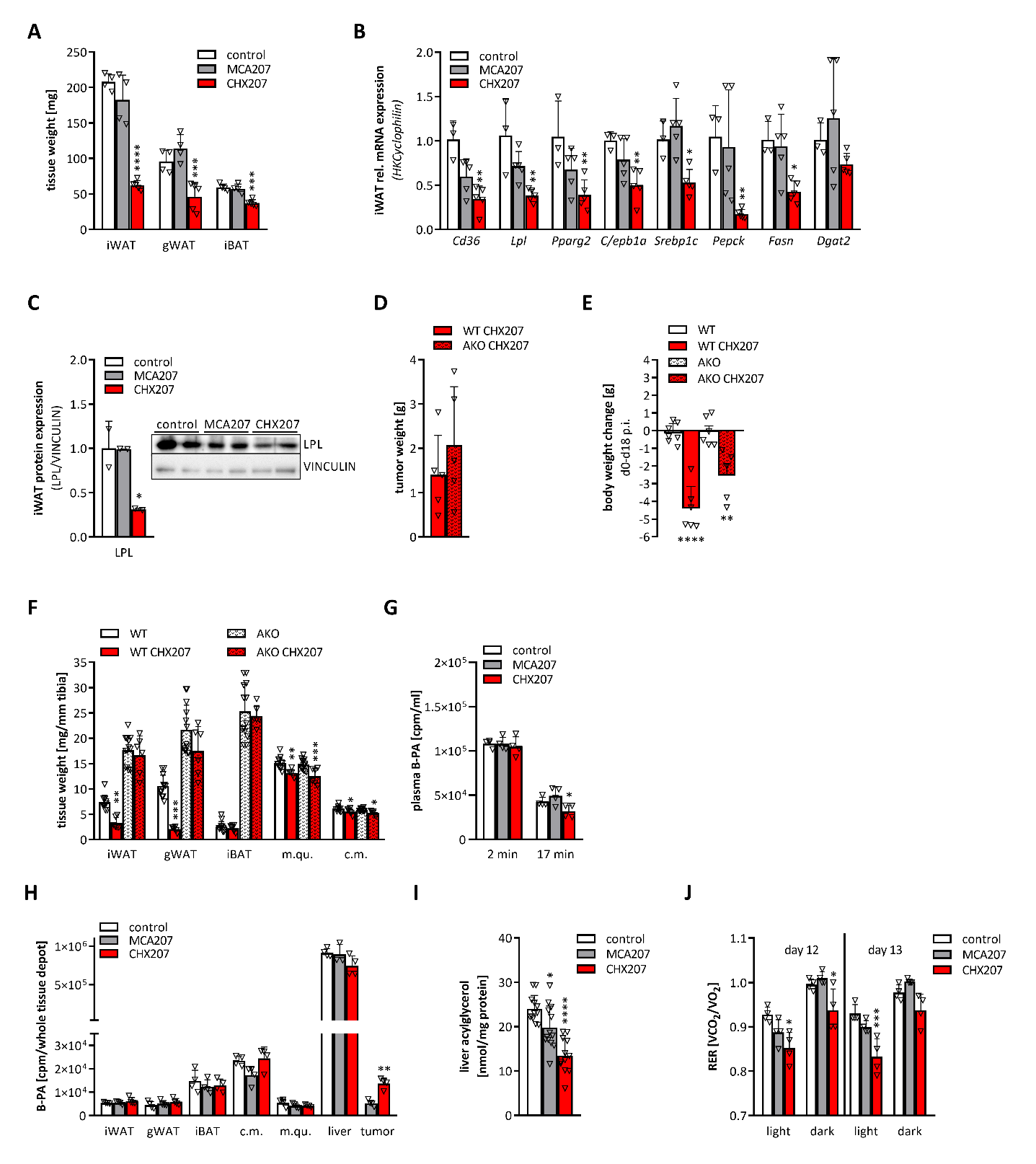


**Figure S4** CHX207 mice exhibit decreased adipolipogenesis, increased FA uptake into tumors, and ATGL deficient mice (AKO/cTg) are protected from CHX207-induced adipose tissue loss. **A-C, G-J)** Ten- to 11-week-old C57BL/6J mice were injected with 1x10^6^ MCA207 cells, 1x10^6^ CHX207 cells, or 1xPBS (control) and were sacrificed with same tumor size (day 15 p.i. for females, day 13 or 15 p.i. for males). **A)** Inguinal subcutaneous (iWAT), gonadal (gWAT) white, and interscapular brown adipose tissue (iBAT) of female C57BL/6J mice were excised and weighed. **B)** mRNA expression levels of marker genes for lipid uptake (*Cd36, Lpl*) and adipogenesis/lipogenesis (*Pparg2, C/ebp1a, Srebp1c, Pepck, Fasn, Dgat2*) in iWAT were determined by qRT-PCR. *Cyclophilin* was used as housekeeping gene (male, day 13 p.i.). **C)** LPL protein expression in iWAT was determined by Western blotting analysis. VINCULIN was used as loading control (male, day 13 p.i.). **D-F)** Fifteen week old male and female wildtype (WT) and ATGL-ko (AKO) mice were injected with 1x10^6^ CHX207 cells or 1xPBS (control) and sacrificed on day 18 p.i.. **D)** Tumor weight (n=5), **E)** body weight change (tumor weight was subtracted) within 18 days (n=6-7), and **F)** weights of iWAT, gWAT, iBAT, and musculus quadriceps (m.qu.) were determined (n=6-10). **G-H)** ^14^C labeled bromo-palmitic acid (B-PA, 1 µCi/mouse, n=4, day 13 p.i.) was intravenously injected and levels of B-PA were measured by liquid scintillation counting in plasma, iWAT, gWAT, iBAT, cardiac muscle (c.m.), musculus quadriceps (m.qu.), livers and tumors. **I)** Total acylglycerol of livers (day 13 p.i., n=12-13) was measured using Infinity Triglycerides Reagent. **J)** Respiratory exchange ratio (RER; VCO_2_/VO_2_) was measured in a laboratory animal monitoring system (n=4, day 12-13 p.i., light and dark phase). Data are presented as means + s.d.. Significance was determined by **A-C,E-J)** one-way ANOVA followed by Tukey’s *post hoc* or **D)** two-sided Student’s t-test analysis (n=3-5,* p ≤ 0.05, **p ≤ 0.01, ***p ≤ 0.001, ****p ≤ 0.0001).





**Figure S5** CHX207 mice exhibit altered blood counts. **A-B)** Ten- to 11-week-old male C57BL/6J mice were injected with 1x10^6^ CHX207 cells, or 1xPBS (control). Cell counts of whole blood from control and tumor-bearing mice were analyzed using an Abacus Hematology analyzer (n=5-11, day 14 p.i.). **A)** Absolute counts of white blood cells subtypes (Neutrophils (Neu), Lymphocytes (Lym), Monocytes (Mono), Eosinophils (Eos), Basophils (Bas)). **B)** Neutrophil to Lymphocyte Ratio (NLR) of absolute cell counts. Data are presented as means + s.d.. Significance was determined by two-sided Student’s t-test (*p ≤ 0.05, **p ≤ 0.01, ***p ≤ 0.001, ****p ≤ 0.0001).

**
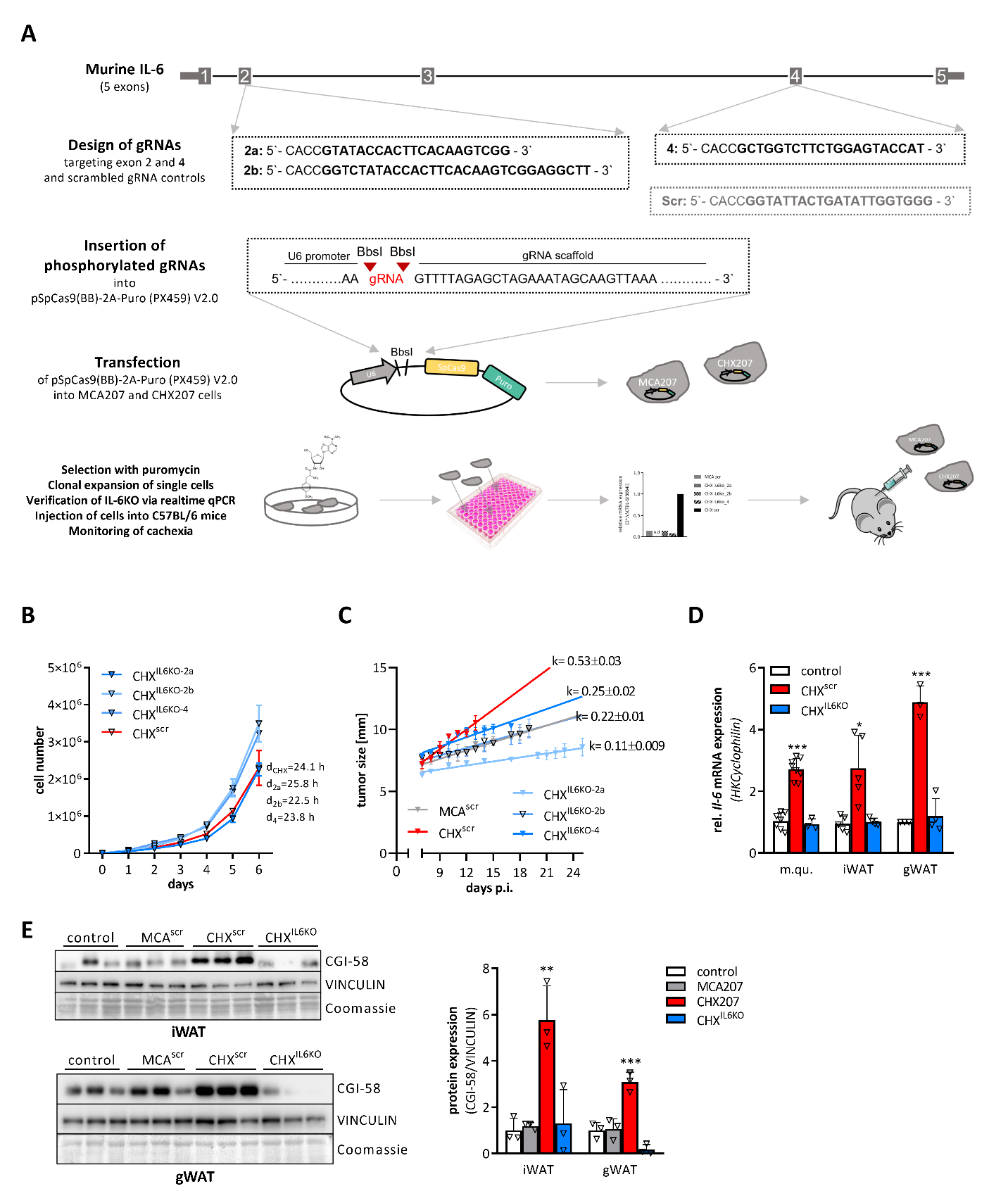
**

**Figure S6** Crispr-Cas9-mediated *Il-6* silencing slows tumor growth and reduces IL-6 signaling in CHX207 mice. **A)** (1) Scheme of the murine *Il-6* gene (NM 031168). (2) Appropriate gRNAs targeting exon 2 (^IL6KO-2a^, ^IL6KO-2b^) and exon 4 (^IL6KO-4^) of *Il-6* for S. pyogenes Cas9 were designed. gRNAs were designed including a 5`overhang for ligation into BbsI sites (5`-CACCG) of the plasmid pSpCas9 (BB) (Addgene #62988). (3) T4 PNK-phosphorylated gRNAs were ligated into BbsI-digested plasmids pSpCas9(BB)-2A-Puro (PX459) V2.0 via Qick Ligase (NEB #M2200). (4) Plasmid-gRNA-constructs were transfected into MCA207 and CHX207 cells via Turbofect™ (Thermo Fisher #R0534). (5) Transfected MCA207 and CHX207 cells were selected with 5 µM puromycin. Puromycin-resistant cells were diluted to single cells and clonal expansion of cells was performed. *Il-6*-knock out of cells was verified by qRT-PCR and 3 different CHX^IL6KO^ cell lines, MCA^scr^ and CHX^scr^ cells were injected into C57BL/6J mice. **B)** Cell proliferation of CHX^scr^, CHX^IL6KO-2a^, CHX^IL6KO-2b^ and CHX^IL6KO-4^ cells in culture was determined by counting the cells for 7 consecutive days after seeding (n=3). **C-E)** Ten- to 11-week-old male C57BL/6J mice were injected with either 1x10^6^ MCAscr, CHXscr, MCA207, CHX207, CHX^IL6KO-2a^, CHX^IL6KO-2b^ or CHX^IL6KO-4^ cells or 1xPBS (control) and sacrificed with same tumor size (1.4 g). **C)** Tumor diameters from day 8 p.i. until day of sacrifice were measured using a sliding caliper and linear regression analysis of tumor growth was performed. **D)** mRNA expression levels of *Il-6* in musculus quadriceps (m.qu.), inguinal white (iWAT), and gonadal white adipose tissue (gWAT) were determined by qRT-PCR. *Cyclophilin* was used as housekeeping gene (n=3-7, day 13 p.i.). **E)** Western blotting analysis and quantification of CGI-58 protein expression in iWAT and gWAT of control and tumor-bearing mice (day 9 p.i.). VINCULIN was used as loading control. Data are presented as means ± s.d.. Significance was determined by one-way ANOVA (n=3-11, *p ≤ 0.05, **p ≤ 0.01, ***p ≤ 0.001).
