# Supplementary material for: Interleukin‐6 initiates muscle‐ and adipose tissue wasting in a novel C57BL/6 model of cancer‐associated cachexia
Source: J Cachexia Sarcopenia Muscle. 2022 Nov 9;14(1):93–107. doi: 10.1002/jcsm.13109 (PMC9891934; doi:10.1002/jcsm.13109)
Supplement: Supplementary file 2 — Table S1. Plasma parameters and cytokine levels of control mice, MCA207 mice, and CHX207 mice. Data are presented as means ± s.d. Significance was determined by two‐sided Student's t‐test (Amino acids) or one‐way ANOVA followed by Tukey's post hoc analysis (n = 4–17, * p ≤ 0.05, **p ≤ 0.01, ***p ≤ 0.001, ****p ≤ 0.0001, n.d. = not detected). [file JCSM-14-93-s002.docx]

**Table 1.** Plasma parameters and cytokine levels of control mice, MCA207 mice, and CHX207 mice. Data are presented as means ± s.d.. Significance was determined by two-sided Student’s t-test (Amino acids) or one-way ANOVA followed by Tukey’s post hoc analysis (n=4-17, * p ≤ 0.05, **p ≤ 0.01, ***p ≤ 0.001, ****p ≤ 0.0001, n.d.= not detected).

| **Unit** | **Plasma parameters** | **control mice** | **MCA207 mice** | **CHX207 mice** |
| --- | --- | --- | --- | --- |
| *mmol/l* | *FA* | 0.50 ± 0.188 | 0.55 ± 0.164 | 0.43 ± 0.180 |
|  | *TG* | 0.695 ± 0.26 | 0.745 ± 0.37 | 0.763 ± 0.25 |
|  | *Glycerol* | 0.198 ± 0.07 | 0.203 ± 0.04 | 0.166 ± 0.05 |
|  | *Lactate* | 3.80 ± 1.454 | 2.70 ± 0.231 | 3.78 ± 0.618 |
| *pg/ml* | *IL-6* | 5.37 ± 8.9 | 7.09 ± 8.52 | 181.53 ± 57.95 **** |
|  | *PTHrP* | 94.68 ± 13.87 | 91.91 ± 36.73 | 372.37 ± 95.24 **** |
|  | *LIF* | 54.2 ± 22.78 | 140.6 ± 48.52 | 110.2 ± 93.20 |
| *ng/ml* | *Myostatin* | 14.6 ± 3.61 | 15.36 ± 1.54 | 14.71 ± 2.57 |
|  | *Metrnl* | n.d. | n.d. | n.d. |
|  | *TNF-α* | n.d. | n.d. | n.d. |
| *mg/ml* | *Glucose* | 2.12 ± 0.41 | 2.16 ± 0.40 | 2.16 ± 0.31 |
|  | *Albumin* | 30.9 ± 1.62 | 29.8 ± 1.53 | 30.4 ± 3.10 |
|  | *Amino acids* | not analyzed | 4164.2 ± 545.8 | 4619.9 ± 813.5 |
| *U/l* | *Creatine kinase* | 216.8±53.9 | 143±54.3 | 395±129.1 |
