# Supplementary material for: Interleukin‐6 initiates muscle‐ and adipose tissue wasting in a novel C57BL/6 model of cancer‐associated cachexia
Source: J Cachexia Sarcopenia Muscle. 2022 Nov 9;14(1):93–107. doi: 10.1002/jcsm.13109 (PMC9891934; doi:10.1002/jcsm.13109)
Supplement: Supplementary file 4 — Data S1. Supplemental References [file JCSM-14-93-s004.docx]

**Supplementary References**

**Interleukin-6 initiates muscle- and adipose tissue wasting in a novel C57BL/6 model of cancer-associated cachexia**

*Journal of Cachexia, Sarcopenia and Muscle*

Isabella Pototschnig, Ursula Feiler, Clemens Diwoky, Paul W. Vesely, Thomas Rauchenwald, Margret Paar, Latifa Bakiri, Laura Pajed, Peter Hofer, Karl Kashofer, Nyamdelger Sukhbaatar, Gabriele Schoiswohl, Thomas Weichhart, Gerald Hoefler, Christoph Bock, Martin Pichler, Erwin F. Wagner, Rudolf Zechner, and Martina Schweiger^#^

# Corresponding author affiliation: Institute of Molecular Biosciences, University of Graz, Graz, Austria

Email: [tina.schweiger@uni-graz.at](mailto:tina.schweiger@uni-graz.at)

Supplemental References

1. Simpson-Herren, L.; Sanford, A.H.; Holmquist, J.P. Cell population kinetics of transplanted and metastatic Lewis lung carcinoma. *Cell Tissue Kinet.* **1974**, *7*, 349–61.
2. Benny Klimek, M.E.; Aydogdu, T.; Link, M.J.; Pons, M.; Koniaris, L.G.; Zimmers, T. a Acute inhibition of myostatin-family proteins preserves skeletal muscle in mouse models of cancer cachexia. *Biochem. Biophys. Res. Commun.* **2010**, *391*, 1548–54.
3. Karnbach, C.; Daws, M.R.; Niemi, E.C.; Nakamura, M.C. Immune Rejection of a Large Sarcoma Following Cyclophosphamide and IL-12 Treatment Requires Both NK and NK T Cells and Is Associated with the Induction of a Novel NK T Cell Population. J. Immunol. 2001, 167, 2569–2576.
4. Rutkowski, P.; Kaminska, J.; Kowalska, M.; Ruka, W.; Steffen, J. Cytokine serum levels in soft tissue sarcoma patients: Correlations with clinico-pathological features and prognosis. Int. J. Cancer 2002, 100, 463–471.
5. Almuraikhy, S.; Kafienah, W.; Bashah, M.; Diboun, I.; Jaganjac, M.; Al-Khelaifi, F.; Abdesselem, H.; Mazloum, N.A.; Alsayrafi, M.; Mohamed-Ali, V.; et al. Interleukin-6 induces impairment in human subcutaneous adipogenesis in obesity-associated insulin resistance. Diabetologia 2016, 59, 2406–2416.
6. Greenberg AS, Nordan RP, McIntosh J, Calvo JC, Scow RO, Jablons D. Interleukin 6 reduces lipoprotein lipase activity in adipose tissue of mice in vivo and in 3T3-L1 adipocytes: a possible role for interleukin 6 in cancer cachexia. Cancer Res 1992;52:4113–6.
