# Supplementary material for: Interleukin‐6 initiates muscle‐ and adipose tissue wasting in a novel C57BL/6 model of cancer‐associated cachexia
Source: J Cachexia Sarcopenia Muscle. 2022 Nov 9;14(1):93–107. doi: 10.1002/jcsm.13109 (PMC9891934; doi:10.1002/jcsm.13109)
Supplement: Supplementary file 5 — Data S2. Supplemental Methods [file JCSM-14-93-s001.docx]

**Supplementary Methods**

**Interleukin-6 initiates muscle- and adipose tissue wasting in a novel C57BL/6 model of cancer-associated cachexia**

*Journal of Cachexia, Sarcopenia and Muscle*

Isabella Pototschnig, Ursula Feiler, Clemens Diwoky, Paul W. Vesely, Thomas Rauchenwald, Margret Paar, Latifa Bakiri, Laura Pajed, Peter Hofer, Karl Kashofer, Nyamdelger Sukhbaatar, Gabriele Schoiswohl, Thomas Weichhart, Gerald Hoefler, Christoph Bock, Martin Pichler, Erwin F. Wagner, Rudolf Zechner, and Martina Schweiger^#^

# Corresponding author affiliation: Institute of Molecular Biosciences, University of Graz, Graz, Austria

Email: [tina.schweiger@uni-graz.at](mailto:tina.schweiger@uni-graz.at)

**Supplementary Methods**

**Animals.**

Mice were bred and maintained under specific pathogen-free conditions at housing temperatures of 21-23°C in a 14 h light/10 h dark cycle and fed a standard chow diet with *ad libitum* access to food and water. Global adipose triglyceride lipase (ATGL)-ko mice expressing ATGL solely in cardiac muscle (AKO) and the respective wild type (WT) control animals were generated as described^1^. All mice used for this study were on a C57Bl/6J background.

**Cell culture.**

MCA207- and CHX207 fibrosarcoma cells were cultured in RPMI 1640 medium (Invitrogen, Waltham, Massachusetts) containing 10% fetal bovine serum (Invitrogen), 100 mg/ml Penicillin/Streptomycin (Invitrogen), 1% Sodium pyruvate MEM (Invitrogen), 1% MEM non essential amino acids (Invitrogen), and 50 µM ß-mercaptoethanol (AppliChem GmbH, Glenview, Illinois). C2C12 myoblasts (#CRL-1772, ATCC, Virginia, USA) were cultured in DMEM medium (4.5g/L glucose, Invitrogen) containing 10% fetal bovine serum (Invitrogen), 100 mg/ml Penicillin/Streptomycin (Invitrogen). Upon confluence C2C12 myoblast cells were differentiated to myotubes by decreasing the concentration of fetal bovine serum (Invitrogen) to 1%^2^**.** Cultured cells were kept at 37 °C, 5% CO_2_, under 95% humidified atmosphere.

**CRISPR/Cas 9-mediated enhancer deletion in cancer cells.**

The CRISPR/Cas 9-mediated enhancer deletion of the *Il6* gene (*Mus musculus*, GenBank: M24221.1) was performed as previously reported^3^. Three gRNAs targeting exon 2 and 4 of *Il-6* and scrambled gRNAs were designed using an online tool ([broadinstitute.org](https://portals.broadinstitute.org/gpp/public/analysis-tools/sgrna-design)). The reverse complement oligos were designed to contain appropriate overhangs for the backbone vector pSpCas9(BB)-2A-Puro (PX459) V2.0 (Addgene, Cambridge, Massachusetts, #62988). The vector was dephosphorylated using Calf Intestinal Alkaline Phosphatase (NEB, #M0290, Ipswich, Massachusetts). The gRNAs were phosphorylated using T4 polynucleotide kinase (NEB, #M0201S), and subsequently ligated into the vector using Quick Ligase (NEB, #M2200). Subsequently the plasmids were transfected into cancer cells using Turbofect™ (Thermo Fisher Scientific) according to manufacturer's instructions. Then, cells were selected using 5 µM puromycin and diluted to 1 cell/96-well. Single cell clones were grown to colonies and *Il-6* deletion was verified by qRT-PCR.

**Treatment with conditioned medium and determination of C2C12 myotube diameter.**

Cancer cells were grown to 80% confluence, medium was replaced by regular growth medium and incubated for 24 h. Thereafter, the medium was collected, centrifuged 288 x g for 5 min at room temperature and filtered using a 3kDa Ultracel membrane (Millipore, Massachusetts, USA). C2C12 myoblasts were cultured in 8-well chamber µ-slides (#80806, ibiTreat-coated, Ibidi, Germany) and differentiated to myotubes as described above. After 5 days, the differentiation medium was replaced with either 1% FCS-containing medium as a control, 10% of MCA207-, or 10% of CHX207-cancer cell conditioned medium. After 48 h cells were washed with 1xPBS and incubated with 4% buffered formaldehyde for 20 min. Thereafter, cells were washed again in 1xPBS and permeabilized with 0.1% Triton X-100 in 1xPBS for 10 min. Afterwards, the cells were washed with 0.1% Tween-20 in 1xPBS (=PBT) for 10 minutes and blocked with 1% BSA at 4°C for 1 h. The α-ACTININ antibody (#3134, Cell signaling) was diluted in 1% BSA in PBT and incubated overnight at 4°C. Cells were washed with PBT four times for 10 min and blocked with 1% BSA in PBT for 15 min. The Alexa Fluor™ 647-labeled secondary antibody (#A21246, Thermo Fisher Scientific) was diluted in 1% BSA in PBT and incubated for 1 h. Thereafter, cells were washed with PBT, covered with 1xPBS and fluorescence microscopy was performed using a Leica SP8 confocal microscope and a Leica FLUOTAR 25x water objective. Background was corrected using a median 3D filter and contrast enhancement was performed allowing 5%-pixel saturation. The image stack was sum projected and diameters of approximately 10 myotubes per field and 20 fields per condition were measured using Fiji.

**Next generation sequencing.**

NGS libraries for mutational screening were prepared using the AmpliSeq library kit 2.0 (Thermo Fisher Scientific) and a custom Ampliseq Primer pool covering the full coding sequence of the murine genes *Cdnk2a*, *Cdkn2d*, *Foxo1*, *Mdm2*, *Myc*, *Pik3ca*, *Pink1*, *Sdhaf1*, *Tert,* and *Trp53*. Sequencing was performed on an Ion Genestudio S5XL benchtop sequencer (Thermo Fisher Scientific) to a length of 400 base pairs (bp) and initial data were analyzed using the Ion Torrent Suite Software Plug-ins (Thermo Fisher Scientific, open source, GPL, <https://github.com/iontorrent/>). Analysis included base calling, alignment to the reference genome (mm10) using the TMAP mapper and variant calling by a modified diBayes approach taking into account the flow space information. Proper PCR amplification of all amplicons and even distribution of NGS reads was documented in coverage analysis plots of each sample. Called variants were annotated using open source software ANNOVAR^4^ and SnpEff^5^. All coding, nonsynonymous mutations were further evaluated and visually inspected in IGV (<http://www.broadinstitute.org/igv/>) and variant calls resulting from technical read errors or sequence effects were excluded from the analysis.

**Transcriptomic analysis.**

Snap-frozen tumor tissue was powdered on dry ice by grinding the frozen tissue fragments in a precooled mortar using a precooled pestle. Tissue powder was transferred to a fresh tube and RNA was isolated using TRIzol reagent according to the manufacturer’s protocol and purified using the RNeasy Mini Kit (QIAGEN, Hilden, Germany). RNA was eluted from RNeasy spin column using RNase-free water. Total RNA was quantified using a Qubit 2.0 Fluorometric Quantitation system (Life Technologies), whereas the RNA integrity number was determined using the Experion Automated Electrophoresis System (Bio-Rad Laboratories, Hercules, USA). RNA-seq was performed using Illumina HiSeq 3000/4000 platform with 50 bp single-end reads to obtain on average 28 million reads per sample. Calling of the bases, using the Illumina Realtime Analysis software, was converted into a BAM format using Illumina2bam and de-multiplexed using BamIndexDecoder (<https://github.com/wtsi-npg/illumina2bam>). Reads were mapped on the mouse reference genome (*Mus musculus*, Ensembl e87) using TopHat2 v.2.0.10. Expression values in graphs are reported as FPKM (fragments per kilobase of transcript per million). Genes are considered expressed when they have FPKM values ≥1. An Excel file giving expression abundance as fragments per kilobase million (FPKM) values for all available genes is provided in Supplemental table 2. Differentially expressed genes (DEGs) between CHX207 and MCA207 tumors were identified by DESeq2^6^. All analyses were performed using R statistics version 4.0.4.

(<https://bioconductor.org/packages/release/bioc/html/EnhancedVolcano.html>).

**Muscle fiber size analysis.**

For muscle fiber size analysis, tissues were prepared as stated in ´Immune-histochemical analysis` and stained with hematoxylin and eosin as described earlier^7^. The resulting slides were scanned and muscle fiber areas (>140 fibers per muscle) were analyzed blinded using a CaseViewer application (3DHISTECH Ltd., Budapest, Hungary).

**MR imaging analysis.**

Mice were anesthetized 3 to 4 min in a chamber containing 4% isoflurane in O_2_. During MRI examination, anesthetic levels were maintained at 1.8-2.0% isoflurane in O_2_ (1 L/min) and respiration rate was kept between 80 and 100 bpm. Mice breathed freely and body temperature was maintained by a water heated animal bed. Mice were scanned in supine position. A balloon pressure sensor (SA Instruments Inc, NY, USA) was placed on the abdomen for respiratory gating. For tumor segmentation a T2-weighed respiratory gated 2D fast spin echo sequence^8^ was used with following parameters: TR/TE=2,829/34 ms, turbo factor=18, slice thickness=500 µm, in-plane resolution=137x137 µm, FOV=35x35 mm, 30 slices. Manual segmentation of the tumor was done in ITK-Snap ^9^. For quantitative R2* mapping a respiratory gated multi-echo gradient sequence was used with parameters: TR=422 ms, 8 monopolar echoes with TE= 1.7/3.1/4.5/5.8/7.2/8.6/9.9/11.3 ms, slice thickness=500 µm, in-plane resolution=225x255 µm, FOV= 45x35 mm, 30 slices. R2* was fitted from the multi-echo data using a variational approach including B0-field correction^10^.

**Metabolic phenotyping.**

Mice were monitored in a laboratory animal monitoring system (PhenoMaster, TSE Systems GmbH, Bad Homburg, Germany) for water intake, O_2_ consumption, CO_2_ production, and activity. Mice were familiarized with drinking flasks for 48 h. Data of the first 12 h of measurement were excluded. Energy expenditure (EE) was calculated using the following equation:

$$E \left[ \frac{kJ}{h} \right]=15.818*V{[O}_{2}]+\frac{5.176*V[{CO}_{2}]}{1000}$$

**Plasma metabolites and cytokines.**

Targeted metabolomics was performed using the Absolute*IDQ*®p180 assay kit (Biocrates Life Sciences AG, Innsbruck, Austria). The fully automated assay was based on PITC (phenylisothiocyanate)-derivatization in the presence of internal standards followed by FIA-MS/MS (acylcarnitines, lipids, and hexoses) and LC/MS (amino acids, biogenic amines) using an AB SCIEX 4000 QTrap® mass spectrometer (AB SCIEX, Darmstadt, Germany) with electrospray ionization^11^.

Plasma concentrations of FA, glycerol, and TG were quantified using NEFA-HR (2) R1 and R2 Set (Wako Chemicals, Neuss, Germany), Free Glycerol Reagent (Sigma Aldrich, St. Louis, Missouri), TG Infinity Reagent (Thermo Fisher Scientific), respectively. Plasma glucose and lactate concentrations were determined using Wellion Calla classic (Med Trust Holding Ges.m.b.H, Marz, Austria) and Accutrend Plus (Cobas, Roche Diagnostics GmbH). Plasma concentrations of IL-6 (#88-7064-22, Invitrogen), PTHrP (#S-1227.0001, Peninsula Laboratories International, Switzerland), LIF (#MLF00, R&D Systems, USA), Myostatin (#K1012, Biomedica, Switzerland), METRNL (#ABIN844457, Antibodies-online, USA) and TNF-a (#88-7324-22, Thermo Fisher) were determined by ELISA.

***In vivo* B-PA uptake studies.**

For uptake studies *in vivo* ^14^C-labeled R-2-bromopalmitic acid (B-PA, #ARC3623, Hartmann Analytic, Germany) was used as tracer^12^. 1 µCi B-PA (dissolved in 100 µl saline) was injected intravenously into the retroorbital plexus of mice that had been fasted for 2 h. Two minutes and 17 min after injection blood was sampled and centrifuged for 8 min at 2300 x g, at 4°C. Seventeen minutes after B-PA injection mice were anesthetized, perfused with 1xPBS (20 ml), sacrificed, and tissues were excised. Tissues were weighed and lysed in 2 ml or 4 ml (liver) SDS/NaOH (0.2%/0.3N) for 3-5 h at 60°C in a shaking water bath. 10 µl of the plasma and 1 ml of the tissue lysates were mixed with Rotiszint eco plus (Roth) and subjected to liquid scintillation counting (Roth; Tri-Carb 2100TR, Packard Instrument Company, Downers Grove, IL).

**Lipolysis of adipose tissue explants.**

Lipolysis assays were performed as previously reported^13^. Gonadal WAT was excised, washed in prewarmed 1xPBS, transferred into DMEM (Gibco-Life Technologies), and carefully cut into fat pads of 1 mm in diameter. For basal lipolysis, fat pads were transferred into DMEM supplemented with 2% FA-free BSA (Sigma Aldrich) and incubated for 60 min at 37 °C, 5% CO2, and 95% humidified atmosphere. For stimulated lipolysis, fat pads were transferred into DMEM supplemented with 2% FA-free BSA and 1 µM isoproterenol (Sigma Aldrich) and incubated for 60 min at same conditions. After incubation, glycerol content in the medium was determined using Free glycerol reagent (Sigma Aldrich). Fat pads were washed in 1xPBS, delipidated using CHCl_3_ extraction for 2 h at 37 °C and solubilized in SDS/NaOH (0.1%/0.3 N) at 65 °C overnight under vigorous shaking. Protein content of fat pads was determined using Pierce BCA Protein assay (Pierce, Thermo Fisher Scientific) and BSA as standard.

**TG hydrolase activity of tissues.**

Snap-frozen tissues were homogenized using Ultra-Turrax Homogenizer (IKA) in ice-cold solution A [0.25 M sucrose, 1 mM EDTA, 1 mM dithiothreitol, pH 7.0] supplemented with protease inhibitors (20 µg/ml leupeptin, 2 µg/ml antipain, 1 µg/ml pepstatin; Carl Roth GmbH, Karlsruhe, Germany)]. Tissue homogenates were centrifuged for 30 min at 4 °C, and 10,000 x g and the fat-free infranatant was collected. Ten µg of homogenate were incubated with a TG substrate. TG substrate was prepared by emulsifying 1.67 mM, 10 µCi/ml triolein [9,10-3H(N)] (NET431L, Perkin Elmer, Waltham, MA), and 190 µM PC/PI (3:1; M:M) in 100 mM potassium phosphate buffer (pH: 7.0) by sonication on ice (Virsonic 475, Virtis, Gardiner, NJ). As FA acceptor 5% FA-free BSA (Sigma Aldrich) was added. Homogenates were incubated with TG substrate for 1 h at 37 °C in a water bath. After incubation, the reaction was terminated by the addition of 650 µl methanol/chloroform/heptane (10/9/7; vol/vol/vol) and 200 µl of 0.1 M potassium-carbonate/0.1 M boric acid (pH: 10.5). The samples were mixed and centrifuged for 10 min at 1,000 x g. An aliquot of 200 µl of the upper aqueous phase was transferred to 2 ml Rotiszint eco plus (Roth). Radioactivity was analyzed using liquid scintillation counting (Roth;Tri-Carb 2100TR, Packard Instrument Company). TG hydrolase activity was determined as released FA per hour and mg protein^13^.

**Tissue acyglycerol content.**

Total lipids were extracted from snap frozen tissues according to Folch et al.^14^. In brief, tissue was homogenized using Ultra-Turrax Homogenizer (IKA, Staufen, Germany) in 1xPBS and extracted using chloroform/methanol (2:1) at 4°C on a spinning wheel. After centrifugation (1,000 g, 10 min) the organic phase was collected, evaporated and dissolved in 2% Triton X-100 by sonication. Acylglycerol levels were determined using TG Infinity Reagent (Thermo Scientific) and glycerol as standard solution. The protein fraction was dried at 60°C overnight and dissolved in 0.3N NaOH/0.1% SDS. Protein concentration was determined using Pierce BCA Protein Assay (Thermo Fisher Scientific, Waltham, MA) and BSA (Thermo Fisher Scientific) as standard.

**Western blotting analysis.**

Tissues were disrupted in ice-cold solution A [0.25 M sucrose, 1 mM EDTA, 1 mM dithiothreitol, pH 7.0] supplemented with protease- and phosphatase inhibitor (phosstop, Sigma Aldrich, St.Louis, USA). Homogenates were centrifuged for 30 min at 4°C and 16,000 x g, the lipid layer was removed, and protein concentration of the infranatant was determined by Protein Assay Dye (Bio-Rad Laboratories, Hercules, USA). After SDS-PAGE, proteins were blotted onto a methanol-activated polyvinylidenfluorid (PVDF) membrane (Carl Roth GmbH) for 1 h at 200 mA. Unspecific binding sites were blocked using 5% milk powder or 5% BSA (Carl Roth GmbH) in 1xTST followed by incubation with a primary and an HRP-conjugated secondary antibody. HRP-conjugated antibodies were detected by chemiluminescence using Clarity Western Enhanced chemiluminescence (ECL) substrate (Bio-Rad Laboratories) and ChemiDoc Touch Imaging System (Bio-Rad Laboratories). Signal intensities were determined by densitometric analyses using Image Lab software (Bio-Rad Laboratories). Specific proteins were detected using following antibodies: P-HSL (Ser660) (#4126, 1:2000, Cell signaling, USA), P-HSL (Ser565) (#4137, 1:2000, Cell signaling), HSL (#4107S, 1:2000, Cell signaling), CGI-58 (#ab59488, 1:1000, Abcam, UK), ATGL (#2138S, 1:2000, Cell signaling), Vinculin (#V9131, 1:20000, Sigma Aldrich), UCP-1 (#14670S, 1:1000, Cell signaling), GAPDH (#2118S, 1:20000, Cell signaling), LPL (1:140, gift from S.G.Young Lab), P-STAT3 (#9145S, 1:1000, Cell signaling), STAT3 (#4904S, 1:1000, Cell signaling), cleaved CASPASE-3 (#9664, Cell signaling), K48-linkage Specific Polyubiquitin (D9D5, #8081, Cell signaling), p62/SQSTM1 (#5114, Cell signaling), and LC3B (#2775, Cell signaling). HRP-linked Anti-rabbit IgG antibody (#A120-201P, 1:10000, Bethyl, USA), HRP-linked Anti-mouse IgG antibody (#NA 931V, 1:10000, GE Healthcare, USA), and HRP-linked Anti-goat IgG (#AP180P, Millipore, USA) were used as secondary antibodies.

**Real-time (RT)-qPCR.**

RT-qPCR was performed using following primers:

*Atrogin1* (fwd: 5`CTTTCAACAGACTGGACTTCTCGA`3, rev: 5`CAGCTCCAACAGCCTTACTACGT`3),

*Bnip3* (fwd: 5`GCTCCCAGACACCACAAGAT`3, rev: 5`TGAGAGTAGCTGTGCGCTTC`3),

*C/ebp1a* (fwd: 5`CAAGAACAGCAACGAGTACCG`3, rev: 5`GTCACTGGTCAACTCCAGCAC`3),

*Cd36* (fwd: 5`GAACCTATTGAAGGCTTACATCC`3, rev: 5`CCCAGTCACTTGTGTTTTGAAC`3),

*Cidea* (fwd: 5`TGCTCTTCTGTATCGCCCAGT`3, rev: 5`GCCGTGTTAAGGAATCTGCTG`3),

*Cpt1b* (fwd: 5`CGAGGATTCTCTGGAACTGC`3, rev: 5`GGTCGCTTCTTCAAGGTCTG`3),

*Cyclophilin* (fwd: 5`GGCTCCGTCGTCTTCCTTTT`3, rev: 5`ACTCGTCCTACAGATTCATCTCC`3),

*Dgat2* (fwd: 5`TTCCTGGCATAAGGCCCTATT`3, rev: 5`AGTCTATGGTGTCTCGGTTGAC`3),

*Fasn* (fwd: 5`TCCTGGAACGAGAACACGATCT`3, rev: 5`GAGACGTGTCACTCCTGGACTTG`3),

*Il6* (fwd: 5`GAGGATACCACTCCCAACAGACC`3, rev: 5`AAGTGCATCATCGTTGTTCATACA`3),

*Lc3b* (fwd: 5`CGGAGCTTTGAACAAAGAGTG`3, rev: 5`TCTCTCACTCTCGTACACTTC`3),

*Lpl* (fwd: 5`TCCAGCCAGGATGCAACA`3, rev: 5` CCACGTCTCCGAGTCCTCTCT`3),

*Myhc* (fwd: 5`AGTCCCAGGTCAACAAGCTG`3, rev: 5`TTTCTCCTGTCACCTCTCAACA`3),

*Mtco1* (fwd: 5`TGCTAGCCGCAGGCATTAC`3, rev: 5`GGGTGCCCAAAGAATCAGAAC`3),

*Murf1* (fwd: 5`AGTGTCCATGTCTGGAGGTCGTTT`3, rev: 5`ACTGGAGCACTCCTGCTTGTAGAT`3),

*Myod* (fwd: 5`ACTTTCTGGAGCCCTCCTGGCA`3, rev: 5` TTTGTTGCACTACACAGCATG`3),

*Myog* (fwd: 5`AGTGAATGCAACTCCCACAG`3, rev: 5`CTGGGAAGGCAACAGACATA`3),

*Ndufv1* (fwd: 5`CTTCCCCACTGGCCTCAAG`3, rev: 5`CCAAAACCCAGTGATCCAGC`3),

*p62* (fwd: 5`TGTGGAACATGGAGGGAAGAG`3, rev: 5` TGTGCCTGTGCTGGAACTTTC`3),

*Pax7* (fwd: 5`GACGACGAGGAAGGAGACAA`3, rev: 5`ACATCTGAGCCCTCATCCAG),

*Pepck* (fwd: 5`CATATGCTGATCCTGGGCATAAC`3, rev: 5`CAAACTTCATCCAGGCAATGTC`3),

*Pgc1a* (fwd: 5`CCCTGCCATTGTTAAGACC`3, rev: 5`TGCTGCTGTTCCTGTTTTC`3),

*Pparg2* (fwd: 5`CCAGAGCATGGTGCCTTCGCT`3, rev: 5`CAGCAACCATTGGGTCAG`3),

*Prdm16* (fwd: 5`CAGCACGGTGAAGCCATTC`3, rev: 5`GCGTGCATCCGCTTGTG`3),

*Srebp1c* (fwd: 5`GTTACTCGAGCCTGCCTTCAGG`3, rev: 5`CAAGCTTTGGACCTGGGTGTG`3),

*Ucp1* (fwd: 5`ACTGCCACACCTCCAGTCATT`3, rev: 5`CAAGCTTTGGACCTGGGTGTG`3).

**Supplemental References**

1. Haemmerle G, Moustafa T, Woelkart G, Büttner S, Schmidt A, van de Weijer T *et al.* ATGL-mediated fat catabolism regulates cardiac mitochondrial function via PPAR-α and PGC-1. *Nat Med* 2011;**17**:1076–85.

2. Watanabe TM, Higuchi S, Kawauchi K, Tsukasaki Y, Ichimura T, Fujita H. Chromatin plasticity as a differentiation index during muscle differentiation of C2C12 myoblasts. *Biochem Biophys Res Commun* 2012;**418**:742–747.

3. Ran FA, Hsu PD, Wright J, Agarwala V, Scott DA, Zhang F. Genome engineering using the CRISPR-Cas9 system. *Nat Protoc* 2013;**8**:2281–2308.

4. Wang K, Li M, Hakonarson H. ANNOVAR: functional annotation of genetic variants from high-throughput sequencing data. *Nucleic Acids Res* 2010;**38**:e164.

5. Cingolani P, Platts A, Wang LL, Coon M, Nguyen T, Wang L *et al.* A program for annotating and predicting the effects of single nucleotide polymorphisms, SnpEff: SNPs in the genome of Drosophila melanogaster strain w1118; iso-2; iso-3. *Fly (Austin)* 2012;**6**:80–92.

6. Anders S, Huber W. Differential expression analysis for sequence count data. *Genome Biol* 2010;**11**:R106.

7. Tamilarasan KP, Temmel H, Das SK, Al Zoughbi W, Schauer S, Vesely PW *et al.* Skeletal muscle damage and impaired regeneration due to LPL-mediated lipotoxicity. *Cell Death Dis* 2012;**3**:e354–e354.

8. Chavhan GB, Babyn PS, Thomas B, Shroff MM, Haacke EM. Principles, Techniques, and Applications of T2*- based MR Imaging and Its Special Applications. *RSNA* 2009;1433–1451.

9. Yushkevich PA, Piven J, Hazlett HC, Smith RG, Ho S, Gee JC *et al.* User-guided 3D active contour segmentation of anatomical structures: Significantly improved efficiency and reliability. *Neuroimage* 2006;**31**:1116–1128.

10. Bredies K, Kunisch K, Pock T. Total generalized variation. *SIAM J Imaging Sci* 2010;**3**:492–526.

11. Zukunft S, Sorgenfrei M, Prehn C, Möller G, Adamski J. Targeted metabolomics of dried blood spot extracts. *Chromatographia* 2013;**76**:1295–1305.

12. Oakes ND, Kjellstedt a, Forsberg GB, Clementz T, Camejo G, Furler SM *et al.* Development and initial evaluation of a novel method for assessing tissue-specific plasma free fatty acid utilization in vivo using (R)-2-bromopalmitate tracer. *J Lipid Res* 1999;**40**:1155–1169.

13. Schweiger M, Eichmann TO, Taschler U, Zimmermann R, Zechner R, Lass A. Measurement of lipolysis. *Methods Enzymol* 2014;**538**:171–93.

14. Folch J, Lees M, Sloane SGH. A simple method for the isolation and purification of total lipides from animal tissues. *J Biol Chem* 1957;**226**:497–509.
